# Supplementary material for: Cross-disorder comparison of brain structures among 4836 individuals with mental disorders and controls utilizing danish population-based clinical MRI scans
Source: Mol Psychiatry. 2026 Apr 2;31(8):4655–63. doi: 10.1038/s41380-026-03577-5 (PMC13364670; doi:10.1038/s41380-026-03577-5)
Supplement: Supplementary file 1 — Supplemental Material [file 41380_2026_3577_MOESM1_ESM.pdf]

Supplementary Material

Supplementary Methods. MRI processing pipeline, inclusion, and exclusion criteria.
Supplementary Figure S1. Sensitivity analysis on the number of unique patients who received a diagnosis either before or after the MRI scan was taken, measured in months, and included patients who were diagnosed within 3 months before or after the date of the MRI.
Supplementary Figure S2. Regions of interest.
Supplementary Figure S3. MRI dataset information.
Supplementary Figure S4. Comparisons of all ROIs for Population A.
Supplementary Figure S5. Comparisons of all ROIs for Population B.
Supplementary Figure S6. Comparison of findings between Mental Disorders Subgroups and Non-Psychiatric Controls, with prior ENIGMA studies for Population A.
Supplementary Figure S7. Comparison of findings between Mental Disorders Subgroups and Non-Psychiatric Controls, with prior ENIGMA studies for Population C.
Supplementary Figure S8. Comparison of findings for high- (≤1.5mm slice thickness) and low-resolution (>1.5mm slice thickness) scans for Population A and Population B.
Supplementary Table S9. Comparison of statistical models used in ENIGMA studies and the present study.
Supplementary Table S10. Site distribution of Mental Disorders (any) patients and Non-Psychiatric Controls.
Supplementary Table S11. Site distribution of Mental Disorders without Dementia and Non-Psychiatric Controls.
Supplementary Table S12. Site distribution of Dementia patients and Non-Psychiatric Controls.
Supplementary Table S13. Site distribution of Substance Use Disorder patients and Non-Psychiatric Controls.
Supplementary Table S14. Site distribution of Schizophrenia Spectrum Disorder patients and Non-Psychiatric Controls.
Supplementary Table S15. Site distribution of Depression patients and Non-Psychiatric Controls.
Supplementary Table S16. Site distribution of Anxiety Disorder patients and Non-Psychiatric Controls.
Supplementary Table S17. Comparison of Mental Disorders and Dementia patients for previously reported ROI differences for Population A.
Supplementary Table S18. Comparison of Mental Disorders and Dementia patients for previously reported ROI differences for Population B.
Supplementary Table S19. Comparison of Mental Disorders and Dementia patients for previously reported ROI differences for Population C.
Supplementary Table S20. Comparison of findings for high- (≤1.5mm slice thickness) and low-resolution (>1.5mm slice thickness) scans for Population A.
Supplementary Table S21. Comparison of findings for high- (≤1.5mm slice thickness) and low-resolution (>1.5mm slice thickness) scans for Population B.
Supplementary Table S22. Comparison of findings for high- (≤1.5mm slice thickness) and low-resolution (>1.5mm slice thickness) scans for Population C.
Supplementary Table S23. Comparisons of all ROIs for Population A.
Supplementary Table S24. Comparisons of all ROIs for Population B.
Supplementary Table S25. Comparisons of all ROIs for Population C.
Supplementary Table S26. Comparison of findings between Mental Disorders Subgroups and Non-Psychiatric Controls, with prior ENIGMA studies for Population A.
Supplementary Table S27. Comparison of findings between Mental Disorders Subgroups and Non-Psychiatric Controls, with prior ENIGMA studies for Population B.
Supplementary Table S28. Comparison of findings between Mental Disorders Subgroups and Non-Psychiatric Controls, with prior ENIGMA studies for Population C.

Supplementary Methods: MRI processing pipeline, inclusion, and exclusion criteria

This section describes how we processed the clinical MRI dataset and selected the inclusion and exclusion criteria.

1) MRI sequence estimation

We assigned every scan to one or multiple of the following categories: Fluid-attenuated inversion recovery (FLAIR), Diffusion- (DWI), Susceptibility- (SWI), T1- (T1), T2-weighted imaging (T2* excluded), as well as other identified sequences. The categorization was performed by checking the DICOM header entry Series Description (0008,103E) for the following substrings:

- T1-weighted imaging: ‘T1’, ‘t1’, ‘BRAVO’, ‘mprage’, ‘MPRAGE’ (49,108 scans);
- T2-weighted imaging: ‘T2’, ‘t2’ (47,679 scans);
- Fluid-attenuated inversion recovery: ‘FLAIR’, ‘flair’, ‘Flair’ (21,322 scans);
- Susceptibility-weighted imaging: ‘SWI’, ‘swi’, ‘SUSCEPTABILITET’ (20,434 scans);
- Diffusion-weighted Imaging: ‘DWI’, ‘dwi’, ‘MUSE’, ‘Diffusion’, ‘DTI’, ‘dti’, (35,521 scans);
- Other identified sequence types: ’vessel scout’, ‘VRT’, ‘csf flow’, ‘WIP’, ‘svs’, ‘SVS’, ‘animation’, ‘ACOM’, ‘Verification’, ‘BA’, ‘BLACK BLOOD’, ‘Batch’, ‘Cerebral Blood Flow’, ‘DISPLAY’, ‘IMPAX Volume’, ‘MYELO’, ‘MI Reading’, ‘MM Oncology Reading’, ‘SCREENSAVE’, ‘SPINE’, ‘3D Saved State - AutoSave’, ‘Screen Save’, ‘MINIP’, ‘Minimum Intensity’, ‘Min Intensity Projection’, ‘Min Intensity ’, ‘Minimum Intensity Projection’, ‘Tra SWIp MinIP’, ‘BOLD’, ‘Loc’, ‘loc’, ‘Scout’, ‘LOC’, ‘lokal’, ‘LOKAL’, ‘B1 Calibration’, ‘calib’, ‘Calib’, ‘cal’, ‘Cal’, ‘CAL’, ‘PDW’, ‘TOF’, ‘ToF’, ‘tof’, ‘angio’, ‘Angio’, ‘ANGIO’, ‘SWAN’, ‘PCA’, ‘pca’, ‘dce’, ‘PC’, ‘pc’, ‘TRANCE’, ‘trance’, ‘mIP’, ‘MIP’, ‘SURVEY’, ‘Survey’, ‘survey’, ‘CEST’, ‘ASL’, ‘asl’, ‘TRACEW’, ‘travew’, ‘STIR’, ‘stir’, ‘ADC’, ‘Apparent Diffusion Coefficient’, ‘adc’, ‘Perfusion Weighted’, ‘T2*’, ‘t2*’ (71,814 scans).

Based on the categorization, the second step was constructing classes used as training and prediction targets. Scans from the T2 category are assigned to the T2 class except those in the FLAIR category, which were then assigned to the FLAIR class. The intersection of FLAIR and T2 categories occurs because FLAIR is a T2-weighted MRI technique modification often called “T2-FLAIR”. Scans in other intersections of categories were assigned to the “Unlabeled” class. We predicted a label (FLAIR, DWI, SWI, T1, T2, or other identified sequence) for every unlabeled scan (N=71,049), after training and validation on the labeled data (N=242,878). As input features, we used non-private DICOM header tags that might be related to the sequence type. Tags with more than half the entries missing are discarded. The remaining tags used for classification are:

- (0018,0081) Echo Time;
- (0018,0080) Repetition Time;
- (0018,1314) Flip Angle;
- (0018,0091) Echo Train Length;
- (0018,0020) Scanning Sequence;
- (0018,0021) Sequence Variant;
- (0018,1318) dBdt;
- (0018,0022) Scan Options;
- (0018,0084) Imaging Frequency;
- (0018,0086) Echo Numbers.

Missing numeric values were replaced by the median of the feature computed over all scans. The scanning sequence (SS), sequence variant (SV) and, scan options (SO), image type (IT) features were one-hot encoded, i.e., every distinct possible entry (e.g., Spin Echo, Inversion Recovery, Magnetization Transfer Contrast, etc) is represented by a binary vector. Entries that occurred in less than 1% of all training samples were dropped. Some entries occurred under multiple tags, e.g., Inversion Recovery was present under the Scan Options and the

Scanning Sequence tag. These were combined with an OR statement to one binary feature. For SV, SS, and SO tags, empty lists were also converted to binary features. The preprocessing results in 74 features (67 binary, 6 continuous, and 1 discrete). Labeled data is split into a train (N=218,590) and a validation set (N=24,288). XGBoost (xgboost, v.2.0.3)[1] was used for classification. To find good hyperparameters, 5-fold cross-validation was performed using Bayesian optimization. For more robust classification performances, tuning and training of the classifier were repeated 5 times with different initializations of the model's parameter, and majority voting was performed to obtain the final classification. In the case of ties, the T1w class was picked. After sequence prediction, we obtained 80,624 T1w, 51,685 T2w, 24,290 FLAIR, 23,406 SWI, 34,383 DWI, and 99,539 "other" scans.

2) MRI Dicom to NIfTI conversion

We converted to NIfTI all the 80,624 T1w DICOM series obtained after the MRI sequence estimation with dcm2niix (v.1.0.20230411)[2]. 75 conversions resulted in an error and the corresponding scans were discarded. When multiple NIfTI scans were obtained from a single DICOM series, the largest converted file was kept for further processing.

3) Recon-all-clinical

We processed each of the 80,549 converted NIfTI T1w scans with recon-all-clinical[3-6] (FreeSurfer v. 7.4.1). 6,444 scans resulted in errors; this is due to a limited field of view of the images, 2D images, or MRI pathologies that result in an error at any step of the pipeline.

4) Automatic Quality Control

Recon-all-clinical provides estimated Dice[7] scores for the following brain structures:[5] white matter, cortex, lateral ventricle, cerebellum, thalamus, hippocampus, amygdala, pallidum, putamen, and brainstem. We considered a segmentation failure when the Dice score of any structure listed above was below 0.65[5]. 11,549 scans were discarded.

All regions of interest (ROIs) of Sections 3.2 and 3.3, normalized for intracranial volume, with a volume or thickness larger or smaller than 2 times the interquartile range were discarded for further automatic quality control. 3,563 scans were discarded. 58,993 scans remained after automatic quality control.

Age and sex distributions were preserved before and after interquartile range–based filtering (mean age: 55.24 vs. 55.23 years; proportion female: 57.0% vs. 57.2%). After applying inclusion and exclusion criteria (defined below), interquartile range–based filtering in Population A reduced the sample from 9,659 mental disorder patient scans to 9,060 scans (6.2% excluded) and from 7,204 non-psychiatric control scans to 6,984 scans (3.1% excluded). The higher exclusion rate for patient scans reflects that multiple scans were often acquired per MRI visit, typically to replace earlier scans of insufficient quality.

5) Inclusion and exclusion criteria

We have the following three populations based on different criteria for inclusion and exclusion. Diagnostic and medication codes were based on ICD-10 Version: 2019[8]:

Population A:

Non-psychiatric Controls:
For non-psychiatric controls, the following criteria were used:

- No psychiatric diagnosis (F00-F99, G30) before or within 3 months after the acquisition of the MRI scan;
- age >= 18 years at the time of the MRI scan;

- No neurological comorbidities:
    - Nervous system: G00-G99, except G560 (Carpal tunnel syndrome) and G562 (Ulnar nerve neuropathy);
    - Cerebrovascular diagnosis: I60-69, E236E;
    - CNS infections: A022C, A066, A17, A229C, A321, A390, A504, A514B, A521A–B, A548A, A80–A89, B003–B004, B010–B011, B020–B021, B050–B051, B060, B261–B262, B375, B451, B582, B602, E236A;
    - Traumatic brain injury: S060-S069;
    - Skull fracture: S020, S021, S027, S029;
    - Malignant cancer: C00-C99;
    - Benign neoplasm in the nervous system: D32-D33;
    - HIV: B20-B24.

Cases with mental disorders:
For patients with mental disorders, the following diagnoses were considered if present in the electronic health record:

- Psychiatric diagnosis: F00-F99, G30
- diagnosis before or within 3 months after the acquisition of the MRI scan;
- age >= 18 years at the time of the MRI scan;

Population B:

Non-psychiatric Controls:
Further exclusion criteria on Population A based on medication history. Specifically, we removed controls having brain medication utilizing the codes starting with N0[3-7], except for N05AD08 (Droperidol, anti-nausea medication) and N07BA01 (Nicotine, aid in quitting smoking).

Cases with mental disorders: Same as Population A.

Population C:

Non-psychiatric Controls: Same as Population B.

Cases with mental disorders:
Further exclusion criteria on Population B based on neurological medication history and neurological comorbidities:

- brain medication starting with N04.
- No neurological comorbidities:
    - Nervous system: G00-G99, except G560 (Carpal tunnel syndrome) and G562 (Ulnar nerve neuropathy);
    - Cerebrovascular diagnosis: I60-69, E236E;
    - CNS infections: A022C, A066, A17, A229C, A321, A390, A504, A514B, A521A–B, A548A, A80–A89, B003–B004, B010–B011, B020–B021, B050–B051, B060, B261–B262, B375, B451, B582, B602, E236A;
    - Traumatic brain injury: S060-S069;
    - Skull fracture: S020, S021, S027, S029;
    - Malignant cancer: C00-C99;
    - Benign neoplasm in the nervous system: D32-D33;
    - HIV: B20-B24.

Supplementary Figure S1: Sensitivity analysis on the number of unique patients who received a diagnosis either before or after the MRI scan was taken, measured in months, and included patients who were diagnosed within 3 months before or after the date of the MRI (The results are based on the most inclusive criteria (Population A)).

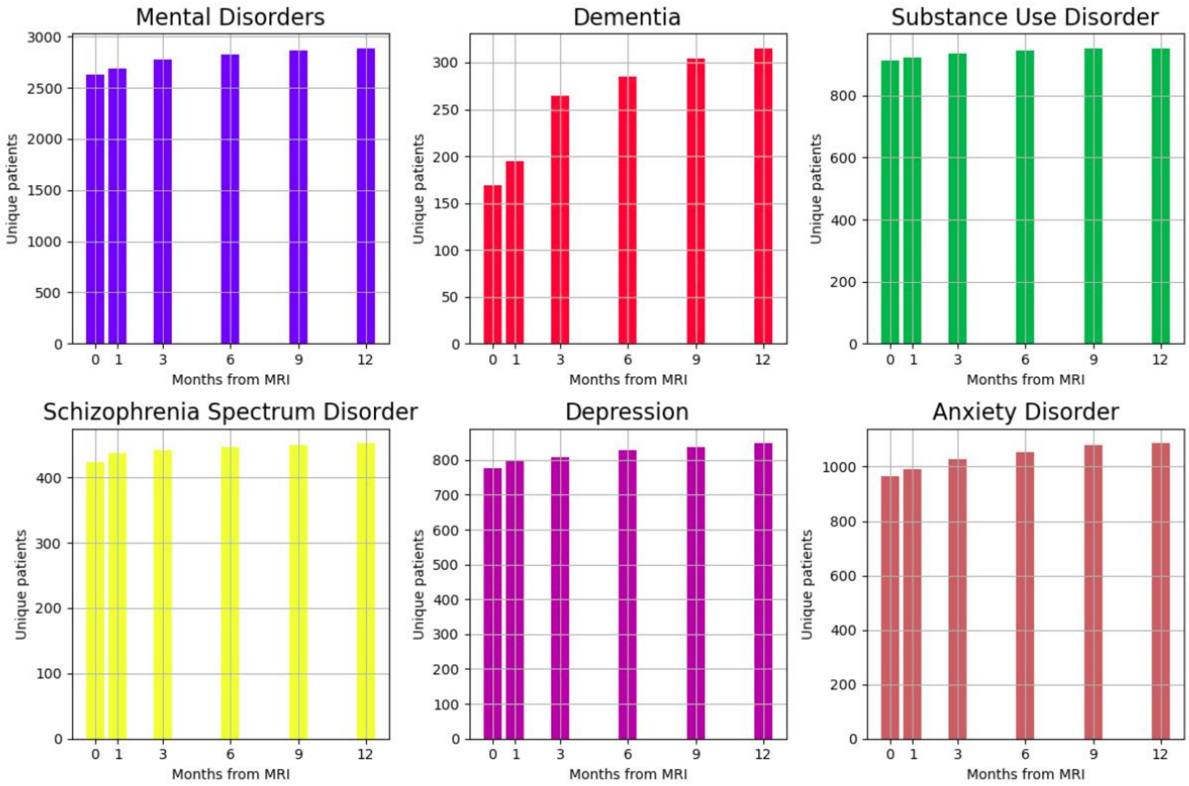

Supplementary Figure S2: Regions of interest. DK atlas parcellation[9] and recon-all-clinical whole-brain segmentation on "fsaverage" from FreeSurfer[10].

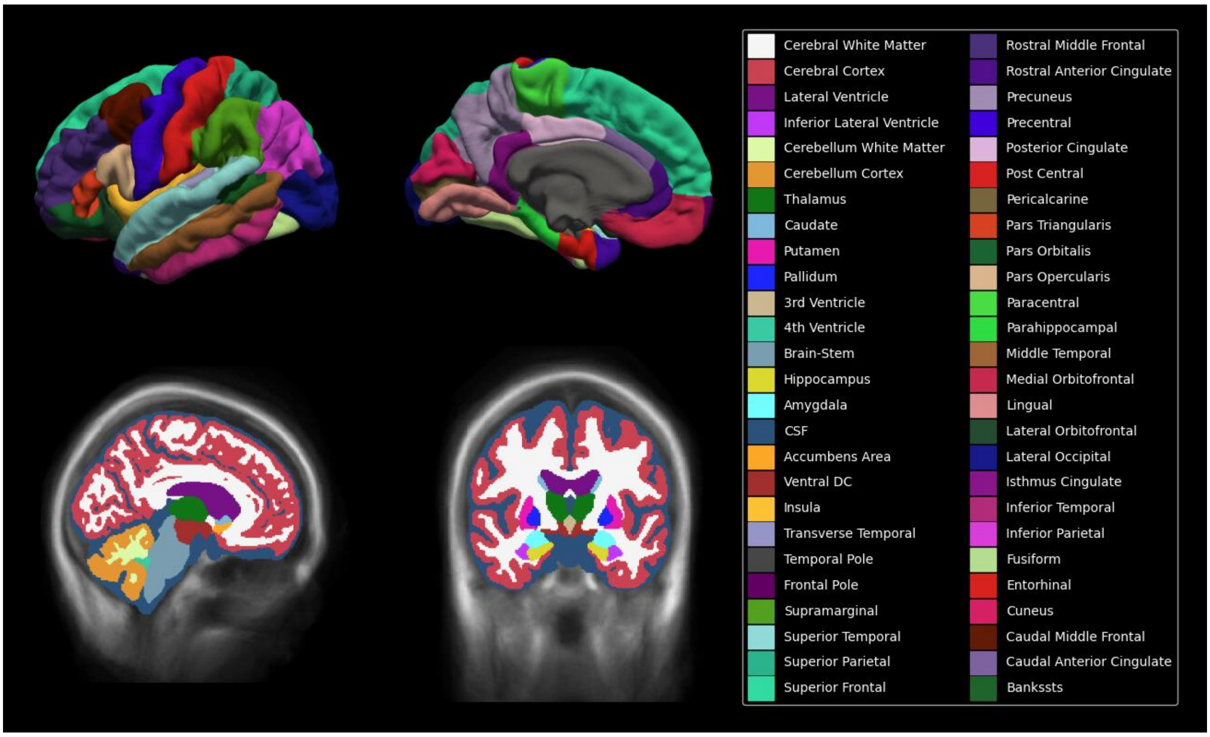

Supplementary Figure S3: MRI dataset information.

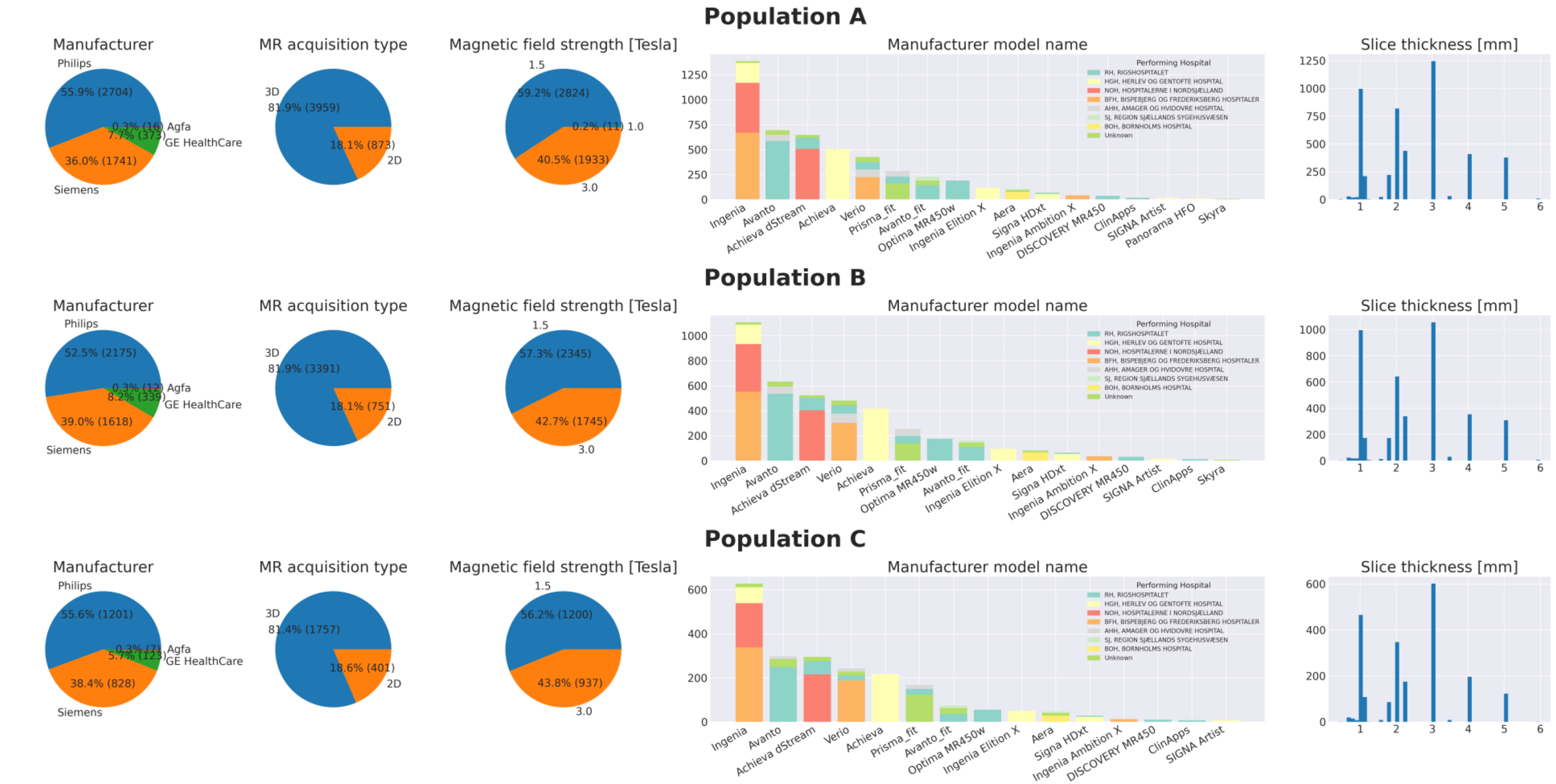

Supplementary Figure S4: Comparisons of all ROIs for Population A. Numerical values are provided in Supplementary Table S23.

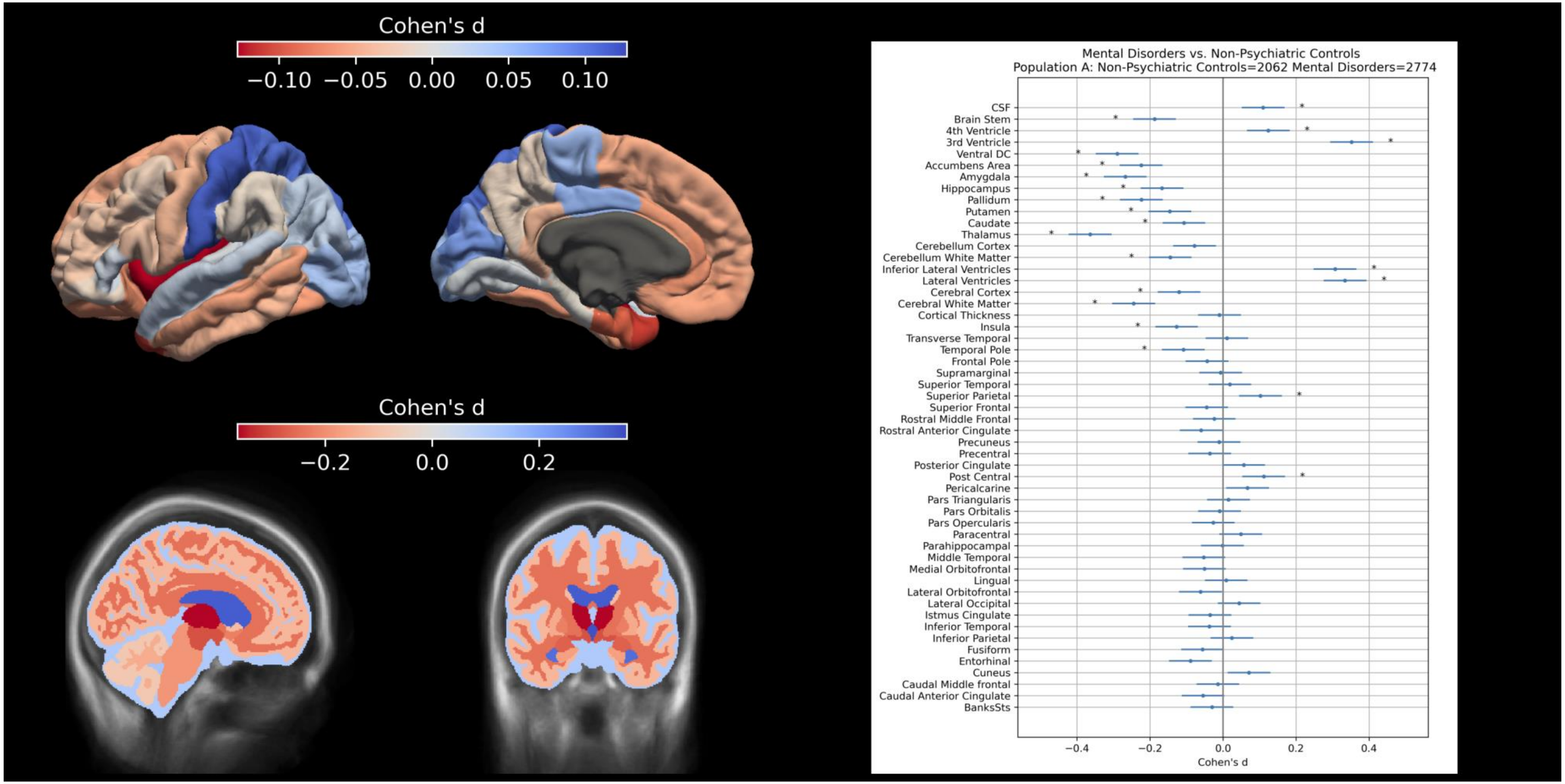

Supplementary Figure S5: Comparisons of all ROIs for Population B. Numerical values are provided in Supplementary Table S24.

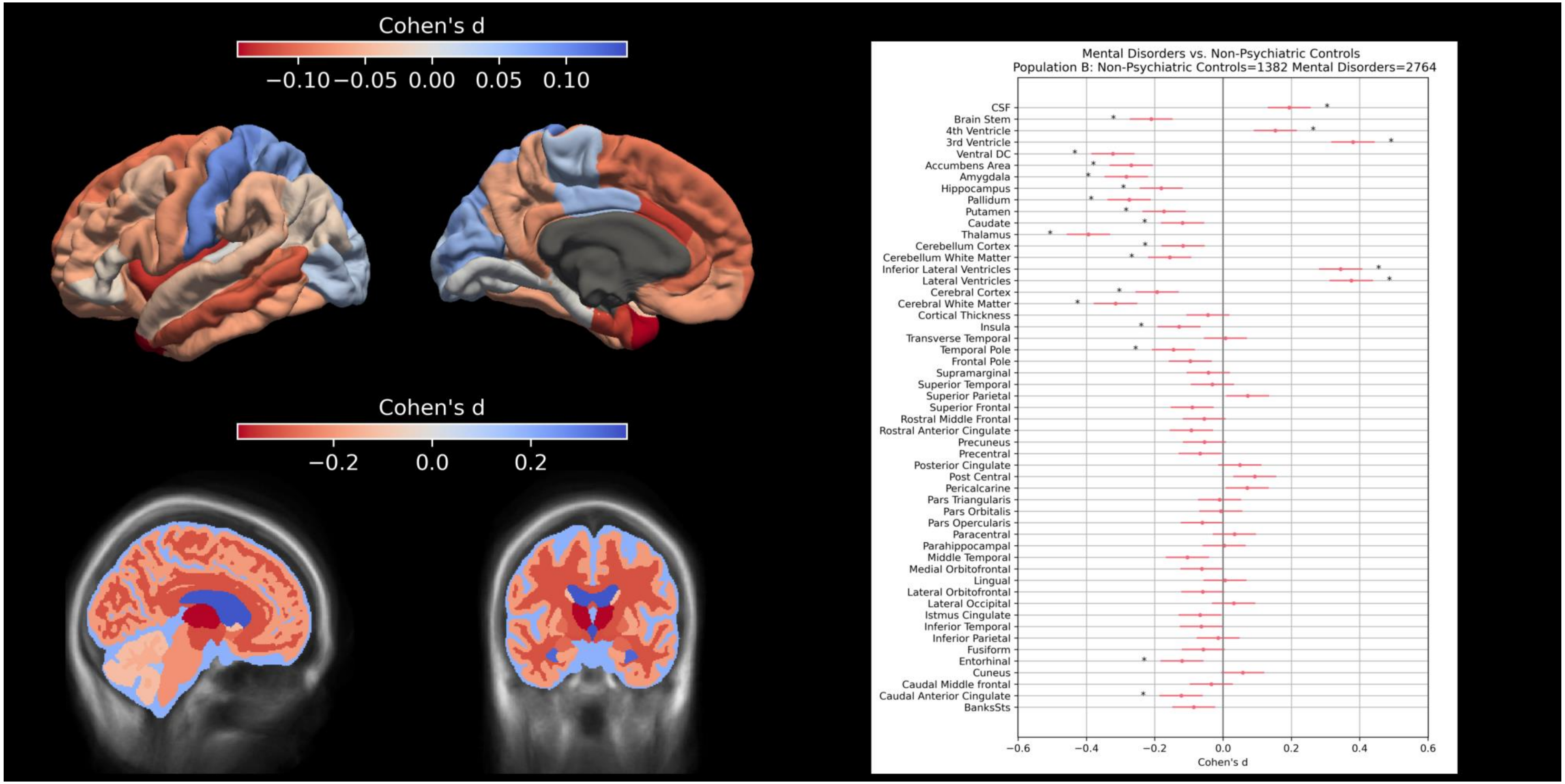

Supplementary Figure S6: Comparison of findings between Mental Disorders Subgroups and Non-Psychiatric Controls, with prior ENIGMA studies for Population A. R=Right hemisphere, L=Left hemisphere, B=Bilateral. Numerical values are provided in Supplementary Table S26.

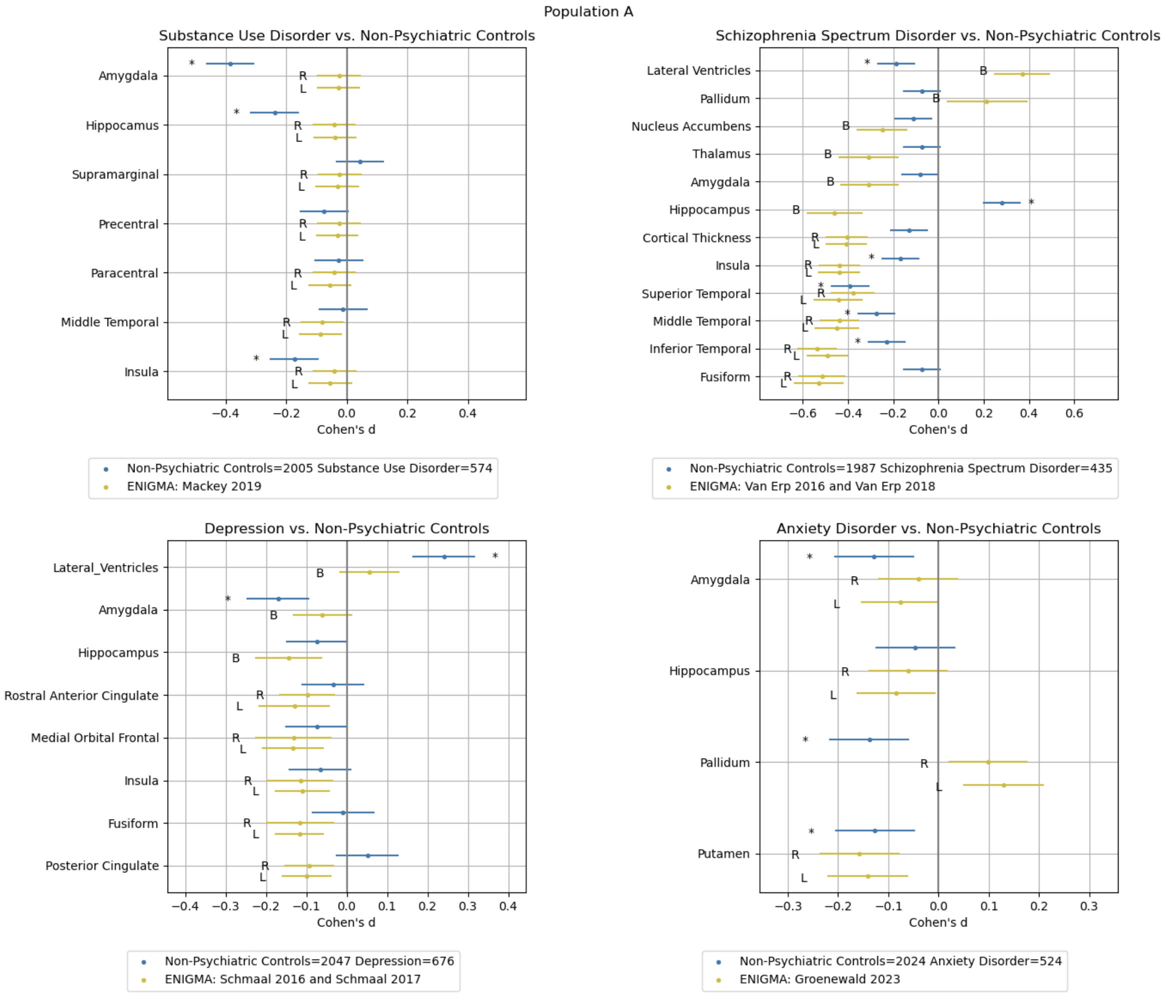

Supplementary Figure S7: Comparison of findings between Mental Disorders Subgroups and Non-Psychiatric Controls, with prior ENIGMA studies for Population C. R=Right hemisphere, L=Left hemisphere, B=Bilateral. Numerical values are provided in Supplementary Table S28.

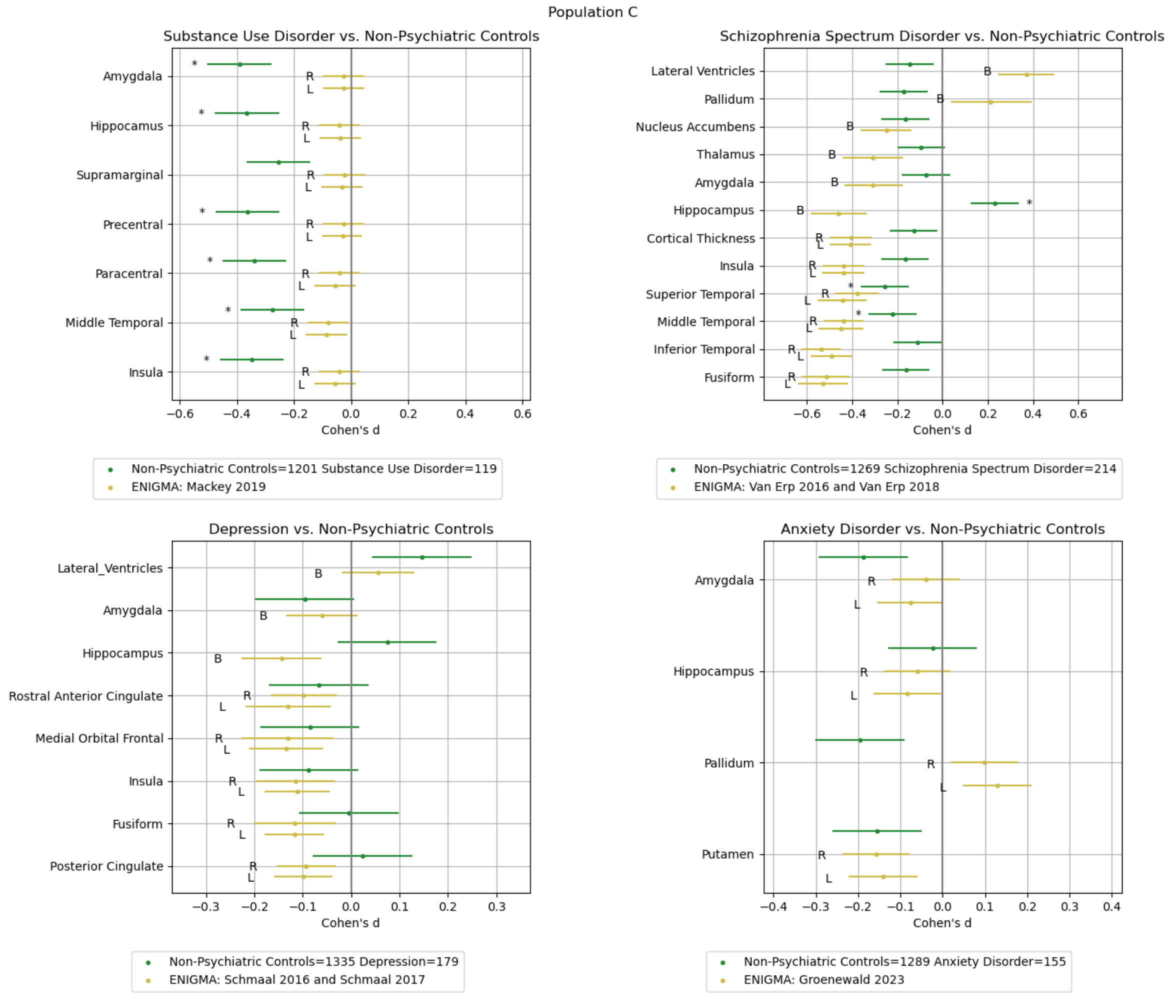

Supplementary Figure S8: Comparison of findings for high- (≤1.5mm slice thickness) and low-resolution (>1.5mm slice thickness) scans for Population A and Population B. Numerical values are provided in Supplementary Table S20 and Table S21.

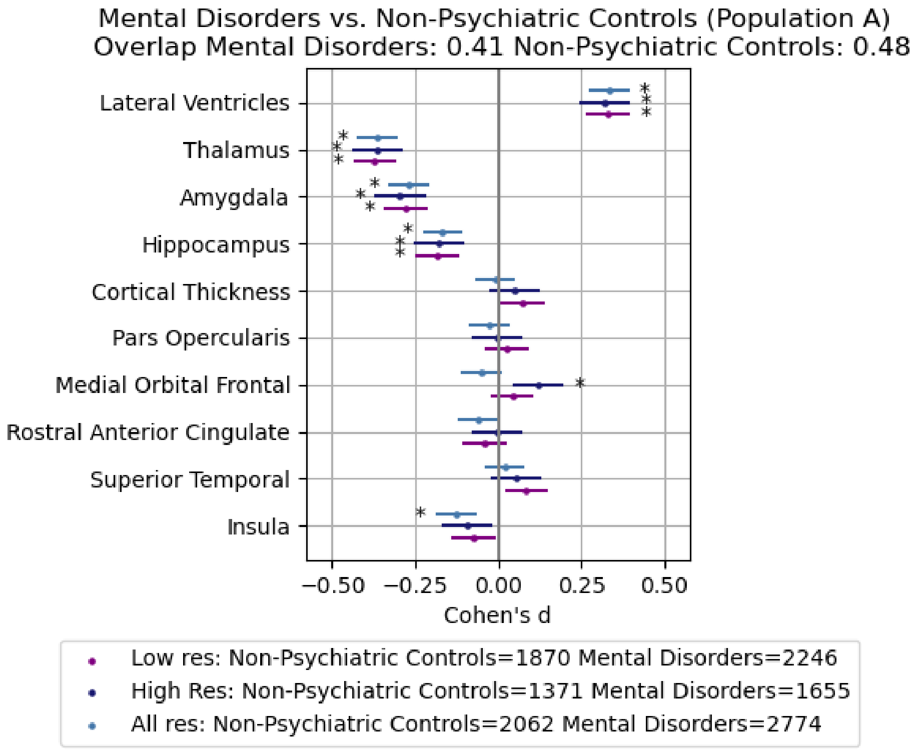

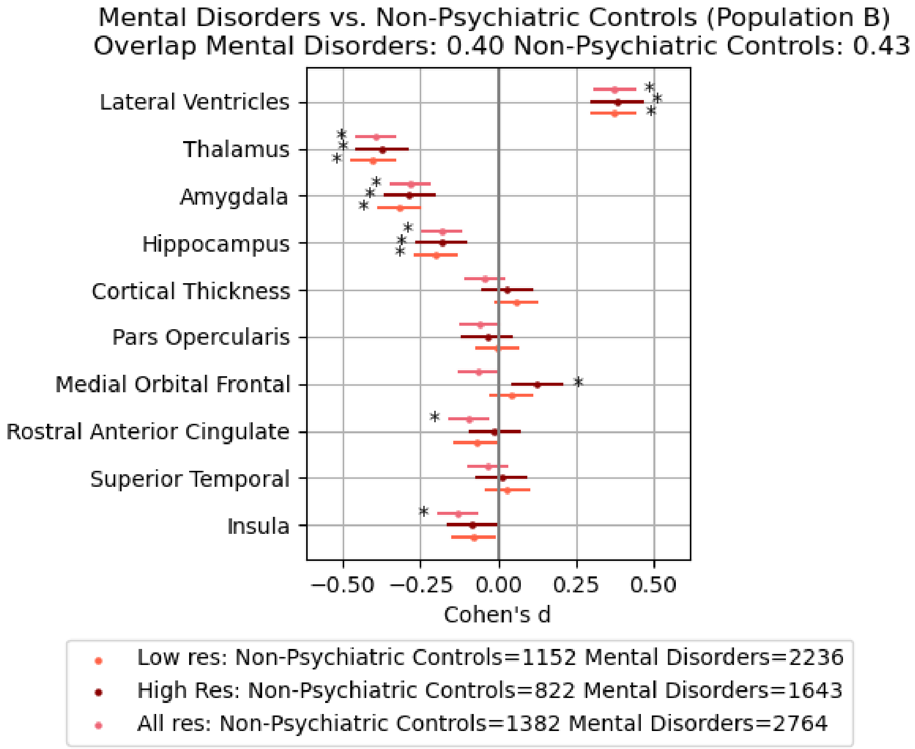

Supplementary Table S9. Comparison of statistical models used in ENIGMA studies and the present study.

ENIGMA study	Model	ROI	Multiple comparisons	Notes
Mackey 2019 [11]	ROI ~ Diagnosis+ Sex + Age + ICV + (1|Site)	Volumes and thicknesses	False discovery rate	Mixed model on Site. ICV for both volumes and thicknesses. "Model" 1 in Mackey 2019.
Van Erp 2016 [12]	ROI ~ Diagnosis + Sex + Age + ICV + Site	Volumes	Bonferroni	"Model 1" in Van Erp 2016.
Van Erp 2018 [13]	ROI ~ Diagnosis + Sex + Age + Site	Thicknesses	False discovery rate	"Model A" in Van Erp 2018.
Schmaal 2016 [14]	ROI ~ Diagnosis + Sex + Age + ICV + Site	Volumes	Bonferroni	None.
Schmaal 2017 [15]	ROI ~ Diagnosis + Sex + Age + Site	Thicknesses	False discovery rate	None.
Groenewold 2023 [16]	ROI ~ Diagnosis + Sex + Age + Age^2 + Sex×Age + Sex×Age^2 + ICV + (1|Site)	Volumes	Family-wise error rate	Mixed model on Site.
Present study	ROI ~ Diagnosis + Sex + Age + [ICV] + Site	Volumes and thicknesses	Bonferroni	ICV only for volumes.

Supplementary Table S10. Site distribution of Mental Disorders (any) patients and Non-Psychiatric Controls. Sites with five or fewer patients are reported as "≤5" to reduce the risk of patient identification.

Site		Population A		Population B		Population C	
**Performing Hospital**	**Manufacturer Model Name**	**Mental Disorders (any)**	**Non-Psychiatric Controls**	**Mental Disorders (any)**	**Non-Psychiatric Controls**	**Mental Disorders (any)**	**Non-Psychiatric Controls**
AHH, AMAGER OG HVIDOVRE HOSPITAL	Avanto	53	9	53	≤5	8	≤5
	Prisma_fit	52	6	52	≤5	16	≤5
	Verio	70	7	70	≤5	10	≤5
BFH, BISPEBJERG OG FREDERIKSBERG HOSPITALER	Ingenia	330	338	330	221	117	221
	Ingenia Ambition X	31	10	31	≤5	8	≤5
	Verio	199	24	199	103	85	103
BOH, BORNHOLMS HOSPITAL	Aera	45	30	45	20	8	20
HGH, HERLEV OG GENTOFTE HOSPITAL	Achieva	293	212	293	124	93	124
	Ingenia	108	92	108	47	26	47
	Ingenia Elition X	66	50	66	28	22	28
	Panorama HFO	10	≤5	-	-	-	-
	SIGNA Artist	11	≤5	11	≤5	≤5	≤5
	Signa HDxt	40	15	40	13	10	13
NOH, HOSPITALERNE I NORDSJÆLLAND	Achieva dStream	256	251	256	146	69	146
	Ingenia	262	237	262	120	80	120
RH, RIGSHOSPITALET	Achieva dStream	58	53	58	42	18	42
	Avanto	382	204	382	152	96	152
	Avanto_fit	88	54	88	21	15	21
	ClinApps	8	8	8	≤5	≤5	≤5
	DISCOVERY MR450	23	12	23	7	≤5	7
	Optima MR450w	145	44	145	31	24	31
	Prisma_fit	42	25	42	18	7	18
	Signa HDxt	≤5	6	≤5	≤5	≤5	≤5
	Verio	48	26	48	17	≤5	17
SJ, REGION SJÆLLANDS SYGEHUSVÆSEN	Aera	≤5	9	≤5	≤5	≤5	≤5
	Avanto_fit	8	31	8	9	≤5	9
Unknown	Achieva dStream	≤5	23	≤5	16	≤5	16
	Aera	≤5	16	≤5	11	≤5	11
	Avanto	15	29	15	26	11	26
	Avanto_fit	12	34	12	22	≤5	22
	Ingenia	≤5	15	≤5	15	≤5	15
	Prisma_fit	31	129	31	106	18	106
	Signa HDxt	≤5	≤5	≤5	≤5	-	-
	Skyra	≤5	≤5	≤5	≤5	-	-
	Verio	24	27	24	15	≤5	15
	Unknown	43	25	43	13	12	13
**TOTAL**		**2774**	**2062**	**2764**	**1382**	**785**	**1376**

Supplementary Table S11. Site distribution of Mental Disorders without Dementia and Non-Psychiatric Controls. Sites with five or fewer patients are reported as "≤5" to reduce the risk of patient identification.

Site		Population A		Population B		Population C	
**Performing Hospital**	**Manufacturer Model Name**	**Mental Disorders without Dementia**	**Non-Psychiatric Controls**	**Mental Disorders without Dementia**	**Non-Psychiatric Controls**	**Mental Disorders without Dementia**	**Non-Psychiatric Controls**
AHH, AMAGER OG HVIDOVRE HOSPITAL	Avanto	52	9	52	≤5	8	≤5
	Prisma_fit	51	6	51	≤5	16	≤5
	Verio	67	7	67	≤5	9	≤5
BFH, BISPEBJERG OG FREDERIKSBERG HOSPITALER	Ingenia	317	338	317	221	116	221
	Ingenia Ambition X	28	10	28	≤5	6	≤5
	Verio	185	24	185	103	83	103
BOH, BORNHOLMS HOSPITAL	Aera	39	30	39	20	8	20
HGH, HERLEV OG GENTOFTE HOSPITAL	Achieva	274	212	274	124	87	124
	Ingenia	101	92	101	47	25	47
	Ingenia Elition X	66	50	66	28	22	28
	Panorama HFO	10	≤5	-	-	-	-
	SIGNA Artist	11	≤5	11	≤5	≤5	≤5
	Signa HDxt	38	15	38	13	9	13
NOH, HOSPITALERNE I NORDSJÆLLAND	Achieva dStream	231	251	231	146	65	146
	Ingenia	242	237	242	120	74	120
RH, RIGSHOSPITALET	Achieva dStream	58	53	58	42	18	42
	Avanto	371	204	371	152	95	152
	Avanto_fit	87	54	87	21	15	21
	ClinApps	6	8	6	≤5	≤5	≤5
	DISCOVERY MR450	22	12	22	7	≤5	7
	Optima MR450w	128	44	128	31	20	31
	Prisma_fit	42	25	42	18	7	18
	Signa HDxt	≤5	6	≤5	≤5	≤5	≤5
	Verio	47	26	47	17	≤5	17
SJ, REGION SJÆLLANDS SYGEHUSVÆSEN	Aera	≤5	9	≤5	≤5	≤5	≤5
	Avanto_fit	8	31	8	9	≤5	9
Unknown	Achieva dStream	≤5	23	≤5	16	≤5	16
	Aera	≤5	16	≤5	11	≤5	11
	Avanto	15	29	15	26	11	26
	Avanto_fit	11	34	11	22	≤5	22
	Ingenia	≤5	15	≤5	15	≤5	15
	Prisma_fit	31	129	31	106	18	106
	Signa HDxt	≤5	≤5	≤5	≤5	-	-
	Skyra	≤5	≤5	≤5	≤5	-	-
	Verio	24	27	24	15	≤5	15
	Unknown	23	25	23	13	8	13
**TOTAL**		**2606**	**2062**	**2596**	**1382**	**751**	**1376**

Supplementary Table S12. Site distribution of Dementia patients and Non-Psychiatric Controls. Sites with five or fewer patients are reported as "≤5" to reduce the risk of patient identification.

Site		Population A		Population B		Population C	
**Performing Hospital**	**Manufacturer Model Name**	**Dementia**	**Non-Psychiatric Controls**	**Dementia**	**Non-Psychiatric Controls**	**Dementia**	**Non-Psychiatric Controls**
AHH, AMAGER OG HVIDOVRE HOSPITAL	Avanto	≤5	9	≤5	≤5	-	-
	Prisma_fit	≤5	6	≤5	≤5	-	-
	Verio	≤5	7	≤5	≤5	≤5	≤5
BFH, BISPEBJERG OG FREDERIKSBERG HOSPITALER	Ingenia	15	338	15	221	≤5	221
	Ingenia Ambition X	≤5	10	≤5	≤5	≤5	≤5
	Verio	15	24	15	103	≤5	103
BOH, BORNHOLMS HOSPITAL	Aera	13	30	13	20	≤5	20
HGH, HERLEV OG GENTOFTE HOSPITAL	Achieva	31	212	31	124	6	124
	Ingenia	7	92	7	47	≤5	47
	Ingenia Elition X	≤5	50	≤5	28	-	-
	Panorama HFO	-	-	-	-	-	-
	SIGNA Artist	-	-	-	-	-	-
	Signa HDxt	≤5	15	≤5	13	≤5	13
NOH, HOSPITALERNE I NORDSJÆLLAND	Achieva dStream	28	251	28	146	6	146
	Ingenia	28	237	28	120	8	120
RH, RIGSHOSPITALET	Achieva dStream	≤5	53	≤5	42	-	-
	Avanto	29	204	29	152	≤5	152
	Avanto_fit	≤5	54	≤5	21	-	-
	ClinApps	≤5	8	≤5	≤5	-	-
	DISCOVERY MR450	≤5	12	≤5	7	-	-
	Optima MR450w	26	44	26	31	7	31
	Prisma_fit	≤5	25	≤5	18	≤5	18
	Signa HDxt	≤5	6	≤5	≤5	-	-
	Verio	≤5	26	≤5	17	-	-
SJ, REGION SJÆLLANDS SYGEHUSVÆSEN	Aera	-	-	-	-	-	-
	Avanto_fit	≤5	31	≤5	9	≤5	9
Unknown	Achieva dStream	-	-	-	-	-	-
	Aera	-	-	-	-	-	-
	Avanto	-	-	-	-	-	-
	Avanto_fit	≤5	34	≤5	22	≤5	22
	Ingenia	≤5	15	≤5	15	-	-
	Prisma_fit	-	-	-	-	-	-
	Signa HDxt	-	-	-	-	-	-
	Skyra	-	-	-	-	-	-
	Verio	-	-	-	-	-	-
	Unknown	41	25	41	13	13	13
**TOTAL**		**262**	**1818**	**262**	**1194**	**60**	**1048**

Supplementary Table S13. Site distribution of Substance Use Disorder patients and Non-Psychiatric Controls. Sites with five or fewer patients are reported as "≤5" to reduce the risk of patient identification.

Site		Population A		Population B		Population C	
**Performing Hospital**	**Manufacturer Model Name**	**Substance Use Disorder**	**Non-Psychiatric Controls**	**Substance Use Disorder**	**Non-Psychiatric Controls**	**Substance Use Disorder**	**Non-Psychiatric Controls**
AHH, AMAGER OG HVIDOVRE HOSPITAL	Avanto	7	9	7	≤5	≤5	≤5
	Prisma_fit	11	6	11	≤5	≤5	≤5
	Verio	≤5	7	≤5	≤5	≤5	≤5
BFH, BISPEBJERG OG FREDERIKSBERG HOSPITALER	Ingenia	76	338	76	221	17	221
	Ingenia Ambition X	≤5	10	≤5	≤5	≤5	≤5
	Verio	41	24	41	103	16	103
BOH, BORNHOLMS HOSPITAL	Aera	≤5	30	≤5	20	-	-
HGH, HERLEV OG GENTOFTE HOSPITAL	Achieva	68	212	68	124	17	124
	Ingenia	28	92	28	47	≤5	47
	Ingenia Elition X	20	50	20	28	≤5	28
	Panorama HFO	≤5	≤5	-	-	-	-
	SIGNA Artist	≤5	≤5	≤5	≤5	≤5	≤5
	Signa HDxt	17	15	17	13	≤5	13
NOH, HOSPITALERNE I NORDSJÆLLAND	Achieva dStream	48	251	48	146	11	146
	Ingenia	67	237	67	120	17	120
RH, RIGSHOSPITALET	Achieva dStream	10	53	10	42	≤5	42
	Avanto	62	204	62	152	≤5	152
	Avanto_fit	15	54	15	21	≤5	21
	ClinApps	≤5	8	≤5	≤5	-	-
	DISCOVERY MR450	≤5	12	≤5	7	-	-
	Optima MR450w	29	44	29	31	≤5	31
	Prisma_fit	8	25	8	18	-	-
	Signa HDxt	≤5	6	≤5	≤5	-	-
	Verio	11	26	11	17	-	-
SJ, REGION SJÆLLANDS SYGEHUSVÆSEN	Aera	≤5	9	≤5	≤5	≤5	≤5
	Avanto_fit	≤5	31	≤5	9	-	-
Unknown	Achieva dStream	-	-	-	-	-	-
	Aera	≤5	16	≤5	11	-	-
	Avanto	-	-	-	-	-	-
	Avanto_fit	≤5	34	≤5	22	≤5	22
	Ingenia	≤5	15	≤5	15	-	-
	Prisma_fit	11	129	11	106	7	106
	Signa HDxt	≤5	≤5	≤5	≤5	-	-
	Skyra	-	-	-	-	-	-
	Verio	≤5	27	≤5	15	-	-
	Unknown	≤5	25	≤5	13	-	-
**TOTAL**		**574**	**2005**	**569**	**1335**	**119**	**1201**

Supplementary Table S14. Site distribution of Schizophrenia Spectrum Disorder patients and Non-Psychiatric Controls. Sites with five or fewer patients are reported as “≤5” to reduce the risk of patient identification.

Site		Population A		Population B		Population C	
**Performing Hospital**	**Manufacturer Model Name**	**Schizo-phrenia Spectrum Disorder**	**Non-Psychiatric Controls**	**Schizo-phrenia Spectrum Disorder**	**Non-Psychiatric Controls**	**Schizo-phrenia Spectrum Disorder**	**Non-Psychiatric Controls**
AHH, AMAGER OG HVIDOVRE HOSPITAL	Avanto	11	9	11	≤5	≤5	≤5
	Prisma_fit	16	6	16	≤5	10	≤5
	Verio	16	7	16	≤5	≤5	≤5
BFH, BISPEBJERG OG FREDERIKSBERG HOSPITALER	Ingenia	56	338	56	221	31	221
	Ingenia Ambition X	≤5	10	≤5	≤5	≤5	≤5
	Verio	48	24	48	103	33	103
BOH, BORNHOLMS HOSPITAL	Aera	≤5	30	≤5	20	≤5	20
HGH, HERLEV OG GENTOFTE HOSPITAL	Achieva	49	212	49	124	25	124
	Ingenia	17	92	17	47	9	47
	Ingenia Elition X	10	50	10	28	6	28
	Panorama HFO	-	-	-	-	-	-
	SIGNA Artist	≤5	≤5	≤5	≤5	≤5	≤5
	Signa HDxt	≤5	15	≤5	13	≤5	13
NOH, HOSPITALERNE I NORDSJÆLLAND	Achieva dStream	24	251	24	146	11	146
	Ingenia	32	237	32	120	12	120
RH, RIGSHOSPITALET	Achieva dStream	19	53	19	42	10	42
	Avanto	66	204	66	152	36	152
	Avanto_fit	12	54	12	21	≤5	21
	ClinApps	-	-	-	-	-	-
	DISCOVERY MR450	≤5	12	≤5	7	-	-
	Optima MR450w	19	44	19	31	6	31
	Prisma_fit	6	25	6	18	≤5	18
	Signa HDxt	-	-	-	-	-	-
	Verio	8	26	8	17	≤5	17
SJ, REGION SJÆLLANDS SYGEHUSVÆSEN	Aera	≤5	9	≤5	≤5	-	-
	Avanto_fit	≤5	31	≤5	9	-	-
Unknown	Achieva dStream	-	-	-	-	-	-
	Aera	-	-	-	-	-	-
	Avanto	≤5	29	≤5	26	≤5	26
	Avanto_fit	≤5	34	≤5	22	-	-
	Ingenia	-	-	-	-	-	-
	Prisma_fit	≤5	129	≤5	106	≤5	106
	Signa HDxt	-	-	-	-	-	-
	Skyra	-	-	-	-	-	-
	Verio	≤5	27	≤5	15	-	-
	Unknown	≤5	25	≤5	13	≤5	13
**TOTAL**		**435**	**1987**	**435**	**1326**	**214**	**1269**

Supplementary Table S15. Site distribution of Depression patients and Non-Psychiatric Controls. Sites with five or fewer patients are reported as "≤5" to reduce the risk of patient identification.

Site		Population A		Population B		Population C	
**Performing Hospital**	**Manufacturer Model Name**	**Depression**	**Non-Psychiatric Controls**	**Depression**	**Non-Psychiatric Controls**	**Depression**	**Non-Psychiatric Controls**
AHH, AMAGER OG HVIDOVRE HOSPITAL	Avanto	13	9	13	≤5	-	-
	Prisma_fit	9	6	9	≤5	≤5	≤5
	Verio	12	7	12	≤5	≤5	≤5
BFH, BISPEBJERG OG FREDERIKSBERG HOSPITALER	Ingenia	70	338	70	221	25	221
	Ingenia Ambition X	9	10	9	≤5	≤5	≤5
	Verio	50	24	50	103	18	103
BOH, BORNHOLMS HOSPITAL	Aera	8	30	8	20	≤5	20
HGH, HERLEV OG GENTOFTE HOSPITAL	Achieva	77	212	77	124	24	124
	Ingenia	27	92	27	47	7	47
	Ingenia Elition X	13	50	13	28	≤5	28
	Panorama HFO	≤5	≤5	-	-	-	-
	SIGNA Artist	≤5	≤5	≤5	≤5	-	-
	Signa HDxt	≤5	15	≤5	13	-	-
NOH, HOSPITALERNE I NORDSJÆLLAND	Achieva dStream	72	251	72	146	19	146
	Ingenia	59	237	59	120	18	120
RH, RIGSHOSPITALET	Achieva dStream	15	53	15	42	≤5	42
	Avanto	103	204	103	152	24	152
	Avanto_fit	20	54	20	21	≤5	21
	ClinApps	≤5	8	≤5	≤5	≤5	≤5
	DISCOVERY MR450	12	12	12	7	≤5	7
	Optima MR450w	36	44	36	31	≤5	31
	Prisma_fit	9	25	9	18	≤5	18
	Signa HDxt	≤5	6	≤5	≤5	≤5	≤5
	Verio	16	26	16	17	≤5	≤5
SJ, REGION SJÆLLANDS SYGEHUSVÆSEN	Aera	-	-	-	-	-	-
	Avanto_fit	≤5	31	≤5	9	≤5	9
Unknown	Achieva dStream	≤5	23	≤5	16	≤5	16
	Aera	≤5	16	≤5	11	≤5	11
	Avanto	≤5	29	≤5	26	≤5	26
	Avanto_fit	≤5	34	≤5	22	≤5	22
	Ingenia	≤5	15	≤5	15	≤5	15
	Prisma_fit	10	129	10	106	≤5	106
	Signa HDxt	-	-	-	-	-	-
	Skyra	-	-	-	-	-	-
	Verio	≤5	27	≤5	15	-	-
	Unknown	≤5	25	≤5	13	≤5	13
**TOTAL**		**676**	**2047**	**674**	**1372**	**179**	**1335**

Supplementary Table S16. Site distribution of Anxiety Disorder patients and Non-Psychiatric Controls. Sites with five or fewer patients are reported as "≤5" to reduce the risk of patient identification.

**Site**		**Population A**		**Population B**		**Population C**	
**Performing Hospital**	**Manufacturer Model Name**	**Anxiety Disorder**	**Non-Psychiatric Controls**	**Anxiety Disorder**	**Non-Psychiatric Controls**	**Anxiety Disorder**	**Non-Psychiatric Controls**
AHH, AMAGER OG HVIDOVRE HOSPITAL	Avanto	12	9	12	≤5	-	-
	Prisma_fit	7	6	7	≤5	≤5	≤5
	Verio	17	7	17	≤5	-	-
BFH, BISPEBJERG OG FREDERIKSBERG HOSPITALER	Ingenia	67	338	67	221	27	221
	Ingenia Ambition X	≤5	10	≤5	≤5	≤5	≤5
	Verio	30	24	30	103	11	103
BOH, BORNHOLMS HOSPITAL	Aera	12	30	12	20	≤5	20
HGH, HERLEV OG GENTOFTE HOSPITAL	Achieva	41	212	41	124	13	124
	Ingenia	19	92	19	47	≤5	47
	Ingenia Elition X	11	50	11	28	≤5	28
	Panorama HFO	-	-	-	-	-	-
	SIGNA Artist	≤5	≤5	≤5	≤5	-	-
	Signa HDxt	8	15	8	13	≤5	13
NOH, HOSPITALERNE I NORDSJÆLLAND	Achieva dStream	53	251	53	146	17	146
	Ingenia	58	237	58	120	22	120
RH, RIGSHOSPITALET	Achieva dStream	7	53	7	42	≤5	42
	Avanto	80	204	80	152	20	152
	Avanto_fit	17	54	17	21	≤5	21
	ClinApps	≤5	8	≤5	≤5	≤5	≤5
	DISCOVERY MR450	≤5	12	≤5	7	≤5	7
	Optima MR450w	22	44	22	31	≤5	31
	Prisma_fit	8	25	8	18	≤5	18
	Signa HDxt	≤5	6	≤5	≤5	-	-
	Verio	7	26	7	17	-	-
SJ, REGION SJÆLLANDS SYGEHUSVÆSEN	Aera	≤5	9	≤5	9	-	-
	Avanto_fit	≤5	31	≤5	≤5	≤5	9
Unknown	Achieva dStream	≤5	23	≤5	16	≤5	16
	Aera	-	-	-	-	-	-
	Avanto	7	29	7	26	6	26
	Avanto_fit	≤5	34	≤5	22	-	-
	Ingenia	-	-	-	-	-	-
	Prisma_fit	8	129	8	106	≤5	106
	Signa HDxt	-	-	-	-	-	-
	Skyra	-	-	-	-	-	-
	Verio	9	27	9	15	≤5	15
	Unknown	≤5	25	≤5	13	≤5	13
**TOTAL**		**524**	**2024**	**524**	**1350**	**155**	**1289**

Supplementary Table S17: Comparison of Mental Disorders and Dementia patients for previously reported ROI differences for Population A.

Population A	Cohen's d [95% CI]	p-value
**Non-Psychiatric Controls (N=2062) vs. Mental Disorders (N=2774)**		
Lateral Ventricles	0.334 [0.278, 0.391]	3.40e-30 *
Thalamus	-0.364 [-0.421, -0.307]	2.23e-35 *
Amygdala	-0.268 [-0.325, -0.211]	4.48e-20 *
Hippocampus	-0.167 [-0.224, -0.111]	9.51e-09 *
Cortical Thickness	-0.010 [-0.066, 0.047]	7.36e-01
Pars Opercularis	-0.027 [-0.083, 0.030]	3.58e-01
Medial Orbitofrontal	-0.051 [-0.108, -0.005]	7.87e-02
Rostral Anterior Cingulate	-0.060 [-0.117, -0.004]	3.85e-02
Superior Temporal	0.019 [-0.037, 0.075]	5.16e-01
Insula	-0.127 [-0.184, -0.071]	1.25e-05 *
**Non-Psychiatric Controls (N=1818) vs. Dementia (N=262)**		
Lateral Ventricles	0.886 [0.795, 0.976]	2.56e-39 *
Hippocampus	-0.603 [-0.691, -0.515]	1.65e-19 *
Cortical Thickness	-0.302 [-0.388, -0.215]	5.33e-06 *
Entorhinal	-0.487 [-0.574, -0.399]	2.57e-13 *
**Non-Psychiatric Controls (N=2062) vs. Mental Disorders without Dementia (N=2606)**		
Lateral Ventricles	0.303 [0.245, 0.361]	1.54e-24 *
Thalamus	-0.353 [-0.411, -0.295]	1.32e-32 *
Amygdala	-0.248 [-0.305, -0.190]	5.46e-17 *
Hippocampus	-0.149 [-0.206, -0.092]	4.46e-07 *
Cortical Thickness	-0.001 [-0.058, 0.056]	9.72e-01
Pars Opercularis	-0.023 [-0.080, 0.035]	4.40e-01
Medial Orbitofrontal	-0.045 [-0.102, 0.013]	1.29e-01
Rostral Anterior Cingulate	-0.056 [-0.114, -0.001]	5.56e-02
Superior Temporal	0.027 [-0.031, 0.084]	3.65e-01
Insula	-0.118 [-0.175, -0.060]	6.76e-05 *

Supplementary Table S18: Comparison of Mental Disorders and Dementia patients for previously reported ROI differences for Population B.

Population B	Cohen's d [95% CI]	p-value
**Non-Psychiatric Controls (N=1382) vs. Mental Disorders (N=2764)**		
Lateral Ventricles	0.376 [0.314, 0.437]	1.18e-29 *
Thalamus	-0.394 [-0.456, -0.333]	1.74e-32 *
Amygdala	-0.283 [-0.344, -0.222]	1.16e-17 *
Hippocampus	-0.181 [-0.242, -0.120]	4.25e-08 *
Cortical Thickness	-0.044 [-0.105, 0.017]	1.78e-01
Pars Opercularis	-0.061 [-0.122, 0.000]	6.53e-02
Medial Orbitofrontal	-0.062 [-0.123, -0.001]	5.88e-02
Rostral Anterior Cingulate	-0.093 [-0.154, -0.032]	4.90e-03 *
Superior Temporal	-0.031 [-0.092, 0.030]	3.44e-01
Insula	-0.129 [-0.190, -0.068]	9.66e-05 *
**Non-Psychiatric Controls (N=1194) vs. Dementia (N=262)**		
Lateral Ventricles	1.037 [0.927, 1.146]	1.84e-48 *
Hippocampus	-0.651 [-0.757, -0.546]	5.80e-21 *
Cortical Thickness	-0.333 [-0.437, -0.230]	1.16e-06 *
Entorhinal	-0.552 [-0.657, -0.448]	1.21e-15 *
**Non-Psychiatric Controls (N=1382) vs. Mental Disorders without Dementia (N=2596)**		
Lateral Ventricles	0.351 [0.289, 0.414]	1.12e-25 *
Thalamus	-0.384 [-0.447, -0.321]	2.72e-30 *
Amygdala	-0.267 [-0.329, -0.204]	1.51e-15 *
Hippocampus	-0.165 [-0.227, -0.103]	7.53e-07 *
Cortical Thickness	-0.037 [-0.099, 0.026]	2.73e-01
Pars Opercularis	-0.058 [-0.120, -0.005]	8.41e-02
Medial Orbitofrontal	-0.056 [-0.118, -0.007]	9.48e-02
Rostral Anterior Cingulate	-0.087 [-0.149, -0.025]	9.07e-03
Superior Temporal	-0.024 [-0.086, 0.038]	4.75e-01
Insula	-0.120 [-0.182, -0.057]	3.36e-04 *

Supplementary Table S19: Comparison of Mental Disorders and Dementia patients for previously reported ROI differences for Population C.

Population C	Cohen's d [95% CI]	p-value
**Non-Psychiatric Controls (N=1376) vs. Mental Disorders (N=785)**		
Lateral Ventricles	0.272 [0.187, 0.357]	1.46e-09 *
Thalamus	-0.298 [-0.383, -0.213]	3.35e-11 *
Amygdala	-0.250 [-0.335, -0.165]	2.54e-08 *
Hippocampus	-0.145 [-0.229, -0.060]	1.25e-03 *
Cortical Thickness	-0.180 [-0.264, -0.095]	5.98e-05 *
Pars Opercularis	-0.158 [-0.242, -0.073]	4.37e-04 *
Medial Orbitofrontal	-0.132 [-0.216, -0.047]	3.24e-03 *
Rostral Anterior Cingulate	-0.124 [-0.208, -0.039]	5.65e-03
Superior Temporal	-0.151 [-0.235, -0.066]	7.62e-04 *
Insula	-0.177 [-0.262, -0.093]	7.67e-05 *
**Non-Psychiatric Controls (N=1048) vs. Dementia (N=60)**		
Lateral Ventricles	0.757 [0.635, 0.879]	1.53e-08 *
Hippocampus	-0.632 [-0.753, -0.512]	2.15e-06 *
Cortical Thickness	-0.648 [-0.769, -0.527]	1.21e-06 *
Entorhinal	-0.690 [-0.811, -0.569]	2.41e-07 *
**Non-Psychiatric Controls (N=1376) vs. Mental Disorders without Dementia (N=751)**		
Lateral Ventricles	0.247 [0.162, 0.333]	5.55e-08 *
Thalamus	-0.283 [-0.369, -0.198]	5.15e-10 *
Amygdala	-0.217 [-0.302, -0.132]	1.89e-06 *
Hippocampus	-0.125 [-0.210, -0.039]	6.11e-03
Cortical Thickness	-0.160 [-0.245, -0.074]	4.46e-04 *
Pars Opercularis	-0.146 [-0.231, -0.060]	1.35e-03 *
Medial Orbitofrontal	-0.122 [-0.207, -0.037]	7.31e-03
Rostral Anterior Cingulate	-0.113 [-0.198, -0.028]	1.26e-02
Superior Temporal	-0.141 [-0.226, -0.055]	1.98e-03 *
Insula	-0.163 [-0.248, -0.078]	3.32e-04 *

Supplementary Table S20: Comparison of findings for high- (≤1.5mm slice thickness) and low-resolution (>1.5mm slice thickness) scans for Population A.

Population A. Overlap: Non-Psychiatric Controls: 47.53% Mental Disorders: 40.62%	All Resolutions: Non-Psychiatric Controls (N=2062) Mental Disorders (N=2774)		High Resolution: Non-Psychiatric Controls (N=1371) Mental Disorders (N=1655)		Low Resolution: Non-Psychiatric Controls (N=1870) Mental Disorders (N=2246)	
	**Cohen's d [95% CI]**	**p-value**	**Cohen's d [95% CI]**	**p-value**	**Cohen's d [95% CI]**	**p-value**
Lateral Ventricles	0.334 [0.278, 0.391]	3.40e-30 *	0.321 [0.250, 0.393]	2.32e-18 *	0.332 [0.270, 0.393]	6.50e-26 *
Thalamus	-0.364 [-0.421, -0.307]	2.23e-35 *	-0.365 [-0.436, -0.293]	4.14e-23 *	-0.371 [-0.433, -0.310]	6.45e-32 *
Amygdala	-0.268 [-0.325, -0.211]	4.48e-20 *	-0.295 [-0.367, -0.224]	8.95e-16 *	-0.278 [-0.339, -0.216]	1.03e-18 *
Hippocampus	-0.167 [-0.224, -0.111]	9.51e-09 *	-0.177 [-0.248, -0.105]	1.40e-06 *	-0.185 [-0.246, -0.123]	4.02e-09 *
Cortical Thickness	-0.010 [-0.066, 0.047]	7.36e-01	0.051 [-0.020, 0.122]	1.63e-01	0.074 [0.013, 0.135]	1.77e-02
Pars Opercularis	-0.027 [-0.083, 0.030]	3.58e-01	0.004 [-0.075, 0.068]	9.18e-01	0.024 [-0.037, 0.085]	4.43e-01
Medial Orbitofrontal	-0.051 [-0.108, -0.005]	7.87e-02	0.122 [0.050, 0.193]	8.77e-04 *	0.043 [-0.018, 0.104]	1.71e-01
Rostral Anterior Cingulate	-0.060 [-0.117, -0.004]	3.85e-02	-0.003 [-0.074, 0.068]	9.35e-01	-0.041 [-0.102, 0.020]	1.88e-01
Superior Temporal	0.019 [-0.037, 0.075]	5.16e-01	0.053 [-0.018, 0.124]	1.45e-01	0.085 [0.024, 0.146]	6.72e-03
Insula	-0.127 [-0.184, -0.071]	1.25e-05 *	-0.092 [-0.163, -0.021]	1.18e-02	-0.074 [-0.135, -0.013]	1.80e-02

Supplementary Table S21: Comparison of findings for high- (≤1.5mm slice thickness) and low-resolution (>1.5mm slice thickness) scans for Population B.

Population B. Overlap: Non-Psychiatric Controls: 42.84% Mental Disorders: 40.38%	All Resolutions: Non-Psychiatric Controls (N=1382) Mental Disorders (N=2764)		High Resolution: Non-Psychiatric Controls (N=822) Mental Disorders (N=1643)		Low Resolution: Non-Psychiatric Controls (N=1152) Mental Disorders (N=2236)	
	**Cohen's d [95% CI]**	**p-value**	**Cohen's d [95% CI]**	**p-value**	**Cohen's d [95% CI]**	**p-value**
Lateral Ventricles	0.376 [0.314, 0.437]	1.18e-29 *	0.383 [0.304, 0.463]	5.70e-19 *	0.372 [0.304, 0.440]	2.73e-24 *
Thalamus	-0.394 [-0.456, -0.333]	1.74e-32 *	-0.374 [-0.453, -0.294]	3.98e-18 *	-0.403 [-0.472, -0.335]	2.96e-28 *
Amygdala	-0.283 [-0.344, -0.222]	1.16e-17 *	-0.286 [-0.366, -0.207]	2.50e-11 *	-0.318 [-0.385, -0.250]	3.18e-18 *
Hippocampus	-0.181 [-0.242, -0.120]	4.25e-08 *	-0.183 [-0.263, -0.104]	1.84e-05 *	-0.202 [-0.270, -0.134]	2.76e-08 *
Cortical Thickness	-0.044 [-0.105, 0.017]	1.78e-01	0.028 [-0.051, 0.107]	5.13e-01	0.057 [-0.011, 0.124]	1.17e-01
Pars Opercularis	-0.061 [-0.122, 0.000]	6.53e-02	-0.035 [-0.114, 0.044]	4.14e-01	-0.003 [-0.071, 0.064]	9.30e-01
Medial Orbitofrontal	-0.062 [-0.123, -0.001]	5.88e-02	0.125 [0.046, 0.204]	3.42e-03 *	0.043 [-0.024, 0.110]	2.35e-01
Rostral Anterior Cingulate	-0.093 [-0.154, -0.032]	4.90e-03 *	-0.011 [-0.090, 0.068]	7.92e-01	-0.071 [-0.138, -0.004]	5.01e-02
Superior Temporal	-0.031 [-0.092, 0.030]	3.44e-01	0.012 [-0.067, 0.091]	7.86e-01	0.030 [-0.038, 0.097]	4.11e-01
Insula	-0.129 [-0.190, -0.068]	9.66e-05 *	-0.083 [-0.162, -0.004]	5.21e-02	-0.080 [-0.147, -0.012]	2.84e-02

Supplementary Table S22: Comparison of findings for high- (≤1.5mm slice thickness) and low-resolution (>1.5mm slice thickness) scans for Population C.

Population C. Overlap: Non-Psychiatric Controls: 42.73% Mental Disorders: 38.98%	All Resolutions: Non-Psychiatric Controls (N=1376) Mental Disorders (N=785)		High Resolution: Non-Psychiatric Controls (N=817) Mental Disorders (N=482)		Low Resolution: Non-Psychiatric Controls (N=1147) Mental Disorders (N=609)	
	Cohen's d [95% CI]	p-value	Cohen's d [95% CI]	p-value	Cohen's d [95% CI]	p-value
Lateral Ventricles	0.272 [0.187, 0.357]	1.46e-09 *	0.319 [0.210, 0.429]	3.30e-08 *	0.245 [0.151, 0.339]	1.13e-06 *
Thalamus	-0.298 [-0.383, -0.213]	3.35e-11 *	-0.281 [-0.391, -0.172]	1.08e-06 *	-0.298 [-0.392, -0.204]	3.37e-09 *
Amygdala	-0.250 [-0.335, -0.165]	2.54e-08 *	-0.283 [-0.392, -0.173]	9.66e-07 *	-0.271 [-0.365, -0.177]	7.40e-08 *
Hippocampus	-0.145 [-0.229, -0.060]	1.25e-03 *	-0.144 [-0.253, -0.035]	1.22e-02	-0.180 [-0.274, -0.086]	3.40e-04 *
Cortical Thickness	-0.180 [-0.264, -0.095]	5.98e-05 *	-0.136 [-0.245, -0.027]	1.81e-02	-0.082 [-0.176, 0.012]	1.02e-01
Pars Opercularis	-0.158 [-0.242, -0.073]	4.37e-04 *	-0.129 [-0.238, -0.020]	2.49e-02	-0.092 [-0.186, 0.002]	6.66e-02
Medial Orbitofrontal	-0.132 [-0.216, -0.047]	3.24e-03 *	0.021 [-0.087, 0.130]	7.10e-01	-0.020 [-0.114, 0.074]	6.91e-01
Rostral Anterior Cingulate	-0.124 [-0.208, -0.039]	5.65e-03	-0.102 [-0.211, 0.007]	7.65e-02	-0.073 [-0.167, 0.021]	1.46e-01
Superior Temporal	-0.151 [-0.235, -0.066]	7.62e-04 *	-0.123 [-0.232, -0.014]	3.22e-02	-0.086 [-0.180, 0.007]	8.52e-02
Insula	-0.177 [-0.262, -0.093]	7.67e-05 *	-0.148 [-0.257, -0.039]	1.03e-02	-0.093 [-0.187, 0.000]	6.32e-02

Supplementary Table S23: Comparisons of all ROIs for Population A.

Population A	Cohen's d [95% CI]	p-value
**Non-Psychiatric Controls (N=2062) vs. Mental Disorders (N=2774)**		
CSF	0.110 [0.054, 0.166]	1.55e-04 *
Brain Stem	-0.188 [-0.244, -0.131]	1.22e-10 *
4th Ventricle	0.124 [0.068, 0.181]	2.01e-05 *
3rd Ventricle	0.352 [0.296, 0.409]	2.59e-33 *
Ventral DC	-0.290 [-0.347, -0.233]	3.37e-23 *
Nucleus Accumbens	-0.224 [-0.281, -0.168]	1.43e-14 *
Amygdala	-0.268 [-0.325, -0.211]	4.48e-20 *
Hippocampus	-0.167 [-0.224, -0.111]	9.51e-09 *
Pallidum	-0.224 [-0.280, -0.167]	1.72e-14 *
Putamen	-0.146 [-0.202, -0.089]	5.52e-07 *
Caudate	-0.107 [-0.164, -0.051]	2.31e-04 *
Thalamus	-0.364 [-0.421, -0.307]	2.23e-35 *
Cerebellum Cortex	-0.078 [-0.134, -0.022]	7.27e-03
Cerebellum White Matter	-0.145 [-0.201, -0.088]	6.87e-07 *
Inferior Lateral Ventricles	0.307 [0.250, 0.364]	9.33e-26 *
Lateral Ventricles	0.334 [0.278, 0.391]	3.40e-30 *
Cerebral Cortex	-0.120 [-0.177, -0.064]	3.50e-05 *
Cerebral White Matter	-0.245 [-0.302, -0.188]	4.77e-17 *
Cortical Thickness	-0.010 [-0.066, 0.047]	7.36e-01
Insula	-0.127 [-0.184, -0.071]	1.25e-05 *
Transverse Temporal	0.011 [-0.046, 0.067]	7.12e-01
Temporal Pole	-0.109 [-0.165, -0.052]	1.84e-04 *
Frontal Pole	-0.044 [-0.100, 0.013]	1.32e-01
Supramarginal	-0.006 [-0.063, 0.050]	8.26e-01
Superior Temporal	0.019 [-0.037, 0.075]	5.16e-01
Superior Parietal	0.103 [0.046, 0.159]	4.15e-04 *
Superior Frontal	-0.045 [-0.101, 0.012]	1.24e-01
Rostral Middle Frontal	-0.024 [-0.080, 0.032]	4.08e-01
Rostral Anterior Cingulate	-0.060 [-0.117, -0.004]	3.85e-02
Precuneous	-0.011 [-0.067, 0.045]	7.08e-01
Precentral	-0.036 [-0.093, 0.020]	2.10e-01
Posterior Cingulate	0.057 [0.000, 0.113]	5.18e-02
Post Central	0.112 [0.055, 0.168]	1.26e-04 *
Pericalcarine	0.067 [0.011, 0.124]	2.09e-02
Pars Triangularis	0.015 [-0.041, 0.071]	6.07e-01
Pars Orbitalis	-0.010 [-0.066, 0.047]	7.40e-01
Pars Opercularis	-0.027 [-0.083, 0.030]	3.58e-01
Paracentral	0.049 [-0.008, 0.105]	9.47e-02
Parahippocampal	-0.002 [-0.058, 0.055]	9.55e-01
Middle Temporal	-0.053 [-0.109, 0.004]	7.06e-02
Medial Orbitofrontal	-0.051 [-0.108, -0.005]	7.87e-02
Lingual	0.009 [-0.048, 0.065]	7.62e-01
Lateral Orbitofrontal	-0.062 [-0.118, -0.005]	3.37e-02
Lateral Occipital	0.044 [-0.012, 0.101]	1.29e-01
Isthmus Cingulate	-0.036 [-0.092, 0.021]	2.19e-01
Inferior Temporal	-0.037 [-0.094, 0.019]	2.01e-01
Inferior Parietal	0.024 [-0.032, 0.081]	4.02e-01
Fusiform	-0.056 [-0.112, 0.000]	5.38e-02
Entorhinal	-0.089 [-0.146, -0.033]	2.18e-03
Cuneous	0.071 [0.015, 0.128]	1.43e-02
Caudal Middle Frontal	-0.014 [-0.070, 0.042]	6.29e-01
Caudal Anterior Cingulate	-0.055 [-0.111, 0.001]	5.86e-02
BanksSts	-0.030 [-0.087, 0.026]	2.97e-01

Supplementary Table S24: Comparisons of all ROIs for Population B.

Population B	Cohen's d [95% CI]	p-value
**Non-Psychiatric Controls (N=1382) vs. Mental Disorders (N=2764)**		
CSF	0.194 [0.133, 0.255]	4.37e-09 *
Brain Stem	-0.210 [-0.271, -0.149]	1.95e-10 *
4th Ventricle	0.153 [0.092, 0.214]	3.48e-06 *
3rd Ventricle	0.381 [0.319, 0.442]	2.11e-30 *
Ventral DC	-0.322 [-0.384, -0.261]	2.29e-22 *
Nucleus Accumbens	-0.269 [-0.330, -0.208]	4.52e-16 *
Amygdala	-0.283 [-0.344, -0.222]	1.16e-17 *
Hippocampus	-0.181 [-0.242, -0.120]	4.25e-08 *
Pallidum	-0.275 [-0.336, -0.214]	9.71e-17 *
Putamen	-0.173 [-0.234, -0.112]	1.65e-07 *
Caudate	-0.118 [-0.179, -0.057]	3.29e-04 *
Thalamus	-0.394 [-0.456, -0.333]	1.74e-32 *
Cerebellum Cortex	-0.117 [-0.178, -0.056]	3.90e-04 *
Cerebellum White Matter	-0.156 [-0.217, -0.095]	2.22e-06 *
Inferior Lateral Ventricles	0.344 [0.283, 0.406]	2.97e-25 *
Lateral Ventricles	0.376 [0.314, 0.437]	1.18e-29 *
Cerebral Cortex	-0.193 [-0.254, -0.132]	5.08e-09 *
Cerebral White Matter	-0.315 [-0.376, -0.253]	2.15e-21 *
Cortical Thickness	-0.044 [-0.105, 0.017]	1.78e-01
Insula	-0.129 [-0.190, -0.068]	9.66e-05 *
Transverse Temporal	0.007 [-0.054, 0.068]	8.32e-01
Temporal Pole	-0.145 [-0.206, -0.084]	1.06e-05 *
Frontal Pole	-0.096 [-0.157, -0.035]	3.62e-03
Supramarginal	-0.043 [-0.104, 0.018]	1.90e-01
Superior Temporal	-0.031 [-0.092, 0.030]	3.44e-01
Superior Parietal	0.072 [0.011, 0.133]	2.81e-02
Superior Frontal	-0.090 [-0.151, -0.029]	6.30e-03
Rostral Middle Frontal	-0.055 [-0.116, -0.006]	9.37e-02
Rostral Anterior Cingulate	-0.093 [-0.154, -0.032]	4.90e-03
Precuneous	-0.054 [-0.115, 0.007]	1.00e-01
Precentral	-0.067 [-0.128, -0.006]	4.15e-02
Posterior Cingulate	0.049 [-0.012, 0.110]	1.37e-01
Post Central	0.093 [0.032, 0.154]	4.69e-03
Pericalcarine	0.071 [0.010, 0.132]	3.16e-02
Pars Triangularis	-0.010 [-0.071, 0.051]	7.62e-01
Pars Orbitalis	-0.007 [-0.068, 0.054]	8.39e-01
Pars Opercularis	-0.061 [-0.122, 0.000]	6.53e-02
Paracentral	0.033 [-0.028, 0.094]	3.12e-01
Parahippocampal	0.003 [-0.058, 0.064]	9.22e-01
Middle Temporal	-0.104 [-0.165, -0.043]	1.59e-03
Medial Orbitofrontal	-0.062 [-0.123, -0.001]	5.88e-02
Lingual	0.006 [-0.055, 0.067]	8.60e-01
Lateral Orbitofrontal	-0.059 [-0.120, 0.002]	7.20e-02
Lateral Occipital	0.031 [-0.030, 0.092]	3.44e-01
Isthmus Cingulate	-0.067 [-0.128, -0.006]	4.15e-02
Inferior Temporal	-0.064 [-0.124, -0.003]	5.40e-02
Inferior Parietal	-0.014 [-0.075, 0.047]	6.65e-01
Fusiform	-0.058 [-0.119, 0.003]	7.81e-02
Entorhinal	-0.120 [-0.181, -0.059]	2.66e-04 *
Cuneous	0.058 [-0.003, 0.119]	7.76e-02
Caudal Middle Frontal	-0.038 [-0.095, 0.027]	2.98e-01
Caudal Anterior Cingulate	-0.123 [-0.184, -0.062]	2.02e-04 *
BanksSts	-0.086 [-0.147, -0.025]	9.24e-03

Supplementary Table S25: Comparisons of all ROIs for Population C.

Population C	Cohen's d [95% CI]	p-value
Non-Psychiatric Controls (N=1376) vs. Mental Disorders (N=785)		
CSF	0.264 [0.179, 0.349]	4.09e-09 *
Brain Stem	-0.153 [-0.237, -0.068]	6.56e-04 *
4th Ventricle	0.049 [-0.035, 0.133]	2.74e-01
3rd Ventricle	0.313 [0.228, 0.398]	3.56e-12 *
Ventral DC	-0.230 [-0.314, -0.145]	3.02e-07 *
Nucleus Accumbens	-0.180 [-0.265, -0.096]	5.82e-05 *
Amygdala	-0.250 [-0.335, -0.165]	2.54e-08 *
Hippocampus	-0.145 [-0.229, -0.060]	1.25e-03
Pallidum	-0.192 [-0.277, -0.108]	1.77e-05 *
Putamen	-0.081 [-0.165, 0.003]	7.03e-02
Caudate	-0.092 [-0.176, -0.008]	3.98e-02
Thalamus	-0.298 [-0.383, -0.213]	3.35e-11 *
Cerebellum Cortex	-0.168 [-0.252, -0.083]	1.81e-04 *
Cerebellum White Matter	-0.149 [-0.233, -0.065]	8.79e-04 *
Inferior Lateral Ventricles	0.351 [0.266, 0.436]	6.65e-15 *
Lateral Ventricles	0.272 [0.187, 0.357]	1.46e-09 *
Cerebral Cortex	-0.225 [-0.310, -0.140]	5.28e-07 *
Cerebral White Matter	-0.246 [-0.331, -0.162]	4.05e-08 *
Cortical Thickness	-0.180 [-0.264, -0.095]	5.98e-05 *
Insula	-0.177 [-0.262, -0.093]	7.67e-05 *
Transverse Temporal	-0.091 [-0.176, -0.007]	4.15e-02
Temporal Pole	-0.234 [-0.318, -0.149]	1.90e-07 *
Frontal Pole	-0.176 [-0.260, -0.091]	8.68e-05 *
Supramarginal	-0.139 [-0.224, -0.055]	1.88e-03
Superior Temporal	-0.151 [-0.235, -0.066]	7.62e-04 *
Superior Parietal	-0.074 [-0.159, 0.010]	9.61e-02
Superior Frontal	-0.212 [-0.297, -0.127]	2.28e-06 *
Rostral Middle Frontal	-0.175 [-0.260, -0.090]	9.41e-05 *
Rostral Anterior Cingulate	-0.124 [-0.208, -0.039]	5.65e-03
Precuneous	-0.180 [-0.265, -0.096]	5.83e-05 *
Precentral	-0.143 [-0.228, -0.059]	1.37e-03
Posterior Cingulate	-0.042 [-0.126, 0.042]	3.48e-01
Post Central	-0.049 [-0.133, 0.036]	2.77e-01
Pericalcarine	-0.005 [-0.089, 0.079]	9.10e-01
Pars Triangularis	-0.110 [-0.194, -0.025]	1.41e-02
Pars Orbitalis	-0.085 [-0.170, -0.001]	5.66e-02
Pars Opercularis	-0.158 [-0.242, -0.073]	4.37e-04 *
Paracentral	-0.070 [-0.155, 0.014]	1.17e-01
Parahippocampal	-0.018 [-0.102, 0.066]	6.89e-01
Middle Temporal	-0.191 [-0.276, -0.107]	1.97e-05 *
Medial Orbitofrontal	-0.132 [-0.216, -0.047]	3.24e-03
Lingual	-0.122 [-0.207, -0.038]	6.31e-03
Lateral Orbitofrontal	-0.121 [-0.206, -0.037]	6.84e-03
Lateral Occipital	-0.098 [-0.182, -0.013]	2.90e-02
Isthmus Cingulate	-0.159 [-0.243, -0.074]	3.89e-04 *
Inferior Temporal	-0.134 [-0.218, -0.049]	2.82e-03
Inferior Parietal	-0.135 [-0.219, -0.050]	2.67e-03
Fusiform	-0.167 [-0.251, -0.082]	1.98e-04 *
Entorhinal	-0.191 [-0.276, -0.107]	1.97e-05 *
Cuneous	-0.063 [-0.147, 0.022]	1.62e-01
Caudal Middle Frontal	-0.139 [-0.223, -0.055]	1.91e-03
Caudal Anterior Cingulate	-0.145 [-0.230, -0.061]	1.18e-03
BanksSts	-0.147 [-0.232, -0.063]	1.02e-03

Supplementary Table S26: Comparison of findings between mental disorders subgroups and controls, with prior ENIGMA studies for Population A. R=Right hemisphere, L=Left hemisphere, B=Bilateral.

Population A	Cohen's d [95% CI]	p-value	ENIGMA studies: Cohen's d [95% CI] [Reference]
**Non-Psychiatric Controls (N=2005) vs. Substance Use Disorder (N=574)**			
Amygdala	-0.385 [-0.463, -0.307]	6.58e-16 *	R: -0.041 [-0.110, 0.028] L: -0.055 [-0.124, 0.014] [11]
Hippocampus	-0.239 [-0.316, -0.161]	4.95e-07 *	R: -0.081 [-0.150, -0.012] L: -0.087 [-0.156, -0.018] [11]
Insula	-0.173 [-0.250, -0.095]	2.67e-04 *	R: -0.042 [-0.111, 0.027] L: -0.056 [-0.125, 0.013] [11]
Middle Temporal	-0.012 [-0.089, 0.065]	7.99e-01	R: -0.026 [-0.095, 0.043] L: -0.030 [-0.099, 0.034] [11]
Paracentral	-0.026 [-0.104, 0.051]	5.77e-01	R: -0.024 [-0.093, 0.045] L: -0.031 [-0.100, 0.038] [11]
Precentral	-0.074 [-0.152, 0.003]	1.17e-01	R: -0.042 [-0.111, 0.027] L: -0.039 [-0.108, 0.030] [11]
Supramarginal	0.043 [-0.034, 0.121]	3.58e-01	R: -0.026 [-0.095, 0.043] L: -0.027 [-0.096, 0.042] [11]
**Non-Psychiatric Controls (N=1987) vs. Schizophrenia Spectrum Disorder (N=435)**			
Hippocampus	0.281 [0.201, 0.361]	1.18e-07 *	B: -0.46 [-0.58, -0.34] [12]
Amygdala	-0.080 [-0.160, 0.000]	1.31e-01	B: -0.31 [-0.43, -0.18] [12]
Thalamus	-0.072 [-0.152, 0.007]	1.72e-01	B: -0.31 [-0.44, -0.18] [12]
Nucleus Accumbens	-0.111 [-0.190, -0.031]	3.65e-02	B: -0.25 [-0.36, -0.14] [12]
Pallidum	-0.074 [-0.154, 0.006]	1.62e-01	B: 0.21 [0.04, 0.39] [12]
Lateral Ventricles	-0.187 [-0.267, -0.107]	4.24e-04 *	B: 0.37 [0.25, 0.49] [12]
Cortical Thickness	-0.132 [-0.211, -0.052]	1.30e-02	R: -0.516 [-0.618, -0.414] L: -0.530 [-0.637, -0.423] [13]
Fusiform	-0.072 [-0.152, 0.007]	1.71e-01	R: -0.536 [-0.62, -0.452] L: -0.491 [-0.579, -0.403] [13]
Inferior Temporal	-0.230 [-0.310, -0.150]	1.48e-05 *	R: -0.439 [-0.522, -0.355] L: -0.449 [-0.543, -0.356] [13]
Middle Temporal	-0.276 [-0.356, -0.196]	2.04e-07 *	R: -0.379 [-0.472, -0.286] L: -0.444 [-0.549, -0.339] [13]
Superior Temporal	-0.391 [-0.471, -0.311]	2.09e-13 *	R: -0.438 [-0.526, -0.351] L: -0.440 [-0.529, -0.352] [13]
Insula	-0.168 [-0.248, -0.089]	1.40e-03 *	R: -0.406 [-0.497, -0.315] L: -0.408 [-0.494, -0.322] [13]
**Non-Psychiatric Controls (N=2047) vs. Depression (N=676)**			
Hippocampus	-0.074 [-0.149, 0.001]	9.51e-02	B: -0.144 [-0.225, -0.064] [14]
Amygdala	-0.170 [-0.245, -0.095]	1.32e-04 *	B: -0.060 [-0.132, 0.011] [14]
Lateral Ventricles	0.241 [0.165, 0.316]	6.31e-08 *	B: 0.056 [-0.017, 0.129] [14]
Posterior Cingulate	0.051 [-0.024, 0.126]	2.51e-01	R: -0.093 [-0.152, -0.034] L: -0.099 [-0.158, -0.040] [15]

Fusiform	-0.009 [-0.084, 0.066]	8.39e-01	R: -0.116 [-0.198, -0.033] L: -0.117 [-0.176, -0.058] [15]
Insula	-0.066 [-0.141, 0.009]	1.39e-01	R: -0.115 [-0.195, -0.035] L: -0.111 [-0.177, -0.045] [15]
Medial Orbitofrontal	-0.075 [-0.150, 0.000]	9.23e-02	R: -0.131 [-0.224, -0.039] L: -0.134 [-0.208, -0.059] [15]
Rostral Anterior Cingulate	-0.034 [-0.109, 0.041]	4.40e-01	R: -0.098 [-0.165, -0.031] L: -0.130 [-0.216, -0.044] [15]
**Non-Psychiatric Controls (N=2024) vs. Anxiety Disorder (N=524)**			
Putamen	-0.127 [-0.205, -0.049]	9.76e-03 *	R: -0.158 [-0.235, -0.080] L: -0.141 [-0.219, -0.062] [16]
Pallidum	-0.138 [-0.216, -0.060]	4.97e-03 *	R: 0.099 [0.021, 0.176] L: 0.129 [0.050, 0.208] [16]
Hippocampus	-0.046 [-0.124, 0.032]	3.49e-01	R: -0.060 [-0.137, 0.016] L: -0.084 [-0.161, -0.007] [16]
Amygdala	-0.129 [-0.206, -0.051]	8.75e-03 *	R: -0.040 [-0.118, 0.037] L: -0.076 [-0.153, -0.001] [16]

Supplementary Table S27: Comparison of findings between mental disorders subgroups and controls, with prior ENIGMA studies for Population B. R=Right hemisphere, L=Left hemisphere, B=Bilateral.

Population B	Cohen's d [95% CI]	p-value	ENIGMA studies: Cohen's d [95% CI] [Reference]
**Non-Psychiatric Controls (N=1335) vs. Substance Use Disorder (N=569)**			
Amygdala	-0.405 [-0.496, -0.314]	1.04e-15 *	R: -0.041 [-0.110, 0.028] L: -0.055 [-0.124, 0.014] [11]
Hippocampus	-0.278 [-0.369, -0.188]	3.13e-08 *	R: -0.081 [-0.150, -0.012] L: -0.087 [-0.156, -0.018] [11]
Insula	-0.188 [-0.278, -0.098]	1.78e-04 *	R: -0.042 [-0.111, 0.027] L: -0.056 [-0.125, 0.013] [11]
Middle Temporal	-0.079 [-0.169, 0.011]	1.15e-01	R: -0.026 [-0.095, 0.043] L: -0.030 [-0.099, 0.034] [11]
Paracentral	-0.055 [-0.145, 0.035]	2.70e-01	R: -0.024 [-0.093, 0.045] L: -0.031 [-0.100, 0.038] [11]
Precentral	-0.117 [-0.207, -0.027]	1.96e-02	R: -0.042 [-0.111, 0.027] L: -0.039 [-0.108, 0.030] [11]
Supramarginal	-0.012 [-0.102, 0.078]	8.13e-01	R: -0.026 [-0.095, 0.043] L: -0.027 [-0.096, 0.042] [11]
**Non-Psychiatric Controls (N=1326) vs. Schizophrenia Spectrum Disorder (N=435)**			
Hippocampus	0.344 [0.250, 0.438]	5.82e-10 *	B: -0.46 [-0.58, -0.34] [12]
Amygdala	-0.055 [-0.148, 0.039]	3.22e-01	B: -0.31 [-0.43, -0.18] [12]
Thalamus	-0.063 [-0.157, 0.030]	2.51e-01	B: -0.31 [-0.44, -0.18] [12]
Nucleus Accumbens	-0.144 [-0.237, -0.050]	9.33e-03	B: -0.25 [-0.36, -0.14] [12]
Pallidum	-0.109 [-0.202, -0.015]	4.94e-02	B: 0.21 [0.04, 0.39] [12]
Lateral Ventricles	-0.169 [-0.263, -0.075]	2.27e-03 *	B: 0.37 [0.25, 0.49] [12]
Cortical Thickness	-0.184 [-0.277, -0.090]	9.13e-04 *	R: -0.516 [-0.618, -0.414] L: -0.530 [-0.637, -0.423] [13]
Fusiform	-0.084 [-0.177, -0.010]	1.29e-01	R: -0.536 [-0.62, -0.452] L: -0.491 [-0.579, -0.403] [13]
Inferior Temporal	-0.217 [-0.311, -0.123]	8.90e-05 *	R: -0.439 [-0.522, -0.355] L: -0.449 [-0.543, -0.356] [13]
Middle Temporal	-0.284 [-0.378, -0.190]	3.11e-07 *	R: -0.379 [-0.472, -0.286] L: -0.444 [-0.549, -0.339] [13]
Superior Temporal	-0.402 [-0.496, -0.307]	5.34e-13 *	R: -0.438 [-0.526, -0.351] L: -0.440 [-0.529, -0.352] [13]
Insula	-0.222 [-0.316, -0.128]	6.15e-05 *	R: -0.406 [-0.497, -0.315] L: -0.408 [-0.494, -0.322] [13]
**Non-Psychiatric Controls (N=1372) vs. Depression (N=674)**			
Hippocampus	-0.094 [-0.181, -0.007]	4.61e-02	B: -0.144 [-0.225, -0.064] [14]
Amygdala	-0.197 [-0.284, -0.110]	2.90e-05 *	B: -0.060 [-0.132, 0.011] [14]
Lateral Ventricles	0.321 [0.233, 0.408]	1.21e-11 *	B: 0.056 [-0.017, 0.129] [14]
Posterior Cingulate	0.044 [-0.043, 0.131]	3.50e-01	R: -0.093 [-0.152, -0.034] L: -0.099 [-0.158, -0.040] [15]
Fusiform	-0.018 [-0.105, 0.069]	7.05e-01	R: -0.116 [-0.198, -0.033] L: -0.117 [-0.176, -0.058] [15]
Insula	-0.063 [-0.150, 0.024]	1.81e-01	R: -0.115 [-0.195, -0.035] L: -0.111 [-0.177, -0.045] [15]

Medial Orbitofrontal	-0.084 [-0.171, 0.003]	7.37e-02	R: -0.131 [-0.224, -0.039] L: -0.134 [-0.208, -0.059] [15]
Rostral Anterior Cingulate	-0.061 [-0.147, 0.026]	1.97e-01	R: -0.098 [-0.165, -0.031] L: -0.130 [-0.216, -0.044] [15]
**Non-Psychiatric Controls (N=1350) vs. Anxiety Disorder (N=524)**			
Putamen	-0.163 [-0.254, -0.072]	1.55e-03 *	R: -0.158 [-0.235, -0.080] L: -0.141 [-0.219, -0.062] [16]
Pallidum	-0.212 [-0.302, -0.121]	4.14e-05 *	R: 0.099 [0.021, 0.176] L: 0.129 [0.050, 0.208] [16]
Hippocampus	-0.066 [-0.157, 0.024]	1.08e-01	R: -0.060 [-0.137, 0.016] L: -0.084 [-0.161, -0.007] [16]
Amygdala	-0.164 [-0.255, -0.073]	1.47e-03 *	R: -0.040 [-0.118, 0.037] L: -0.076 [-0.153, -0.001] [16]

Supplementary Table S28: Comparison of findings between mental disorders subgroups and controls, with prior ENIGMA studies for Population C. R=Right hemisphere, L=Left hemisphere, B=Bilateral.

Population C	Cohen's d [95% CI]	p-value	ENIGMA studies: Cohen's d [95% CI] [Reference]
**Non-Psychiatric Controls (N=1201) vs. Substance Use Disorder (N=119)**			
Amygdala	-0.391 [-0.500, -0.282]	4.91e-05 *	R: -0.041 [-0.110, 0.028] L: -0.055 [-0.124, 0.014] [11]
Hippocampus	-0.365 [-0.474, -0.256]	1.52e-04 *	R: -0.081 [-0.150, -0.012] L: -0.087 [-0.156, -0.018] [11]
Insula	-0.348 [-0.457, -0.239]	3.05e-04 *	R: -0.042 [-0.111, 0.027] L: -0.056 [-0.125, 0.013] [11]
Middle Temporal	-0.275 [-0.384, -0.167]	4.28e-03 *	R: -0.026 [-0.095, 0.043] L: -0.030 [-0.099, 0.034] [11]
Paracentral	-0.338 [-0.447, -0.229]	4.53e-04 *	R: -0.024 [-0.093, 0.045] L: -0.031 [-0.100, 0.038] [11]
Precentral	-0.363 [-0.472, -0.254]	1.65e-04 *	R: -0.042 [-0.111, 0.027] L: -0.039 [-0.108, 0.030] [11]
Supramarginal	-0.254 [-0.363, -0.146]	8.25e-03	R: -0.026 [-0.095, 0.043] L: -0.027 [-0.096, 0.042] [11]
**Non-Psychiatric Controls (N=1269) vs. Schizophrenia Spectrum Disorder (N=214)**			
Hippocampus	0.232 [0.130, 0.334]	1.74e-03 *	B: -0.46 [-0.58, -0.34] [12]
Amygdala	-0.073 [-0.175, 0.029]	3.26e-01	B: -0.31 [-0.43, -0.18] [12]
Thalamus	-0.095 [-0.197, 0.007]	1.98e-01	B: -0.31 [-0.44, -0.18] [12]
Nucleus Accumbens	-0.164 [-0.266, -0.062]	2.69e-02	B: -0.25 [-0.36, -0.14] [12]
Pallidum	-0.172 [-0.274, -0.070]	2.00e-02	B: 0.21 [0.04, 0.39] [12]
Lateral Ventricles	-0.147 [-0.249, -0.045]	4.72e-02	B: 0.37 [0.25, 0.49] [12]
Cortical Thickness	-0.128 [-0.230, -0.027]	8.23e-02	R: -0.516 [-0.618, -0.414] L: -0.530 [-0.637, -0.423] [13]
Fusiform	-0.162 [-0.264, -0.060]	2.83e-02	R: -0.536 [-0.62, -0.452] L: -0.491 [-0.579, -0.403] [13]
Inferior Temporal	-0.110 [-0.212, -0.009]	1.35e-01	R: -0.439 [-0.522, -0.355] L: -0.449 [-0.543, -0.356] [13]
Middle Temporal	-0.222 [-0.324, -0.119]	2.77e-03 *	R: -0.379 [-0.472, -0.286] L: -0.444 [-0.549, -0.339] [13]
Superior Temporal	-0.256 [-0.359, -0.154]	5.35e-04 *	R: -0.438 [-0.526, -0.351] L: -0.440 [-0.529, -0.352] [13]
Insula	-0.166 [-0.268, -0.064]	2.46e-02	R: -0.406 [-0.497, -0.315] L: -0.408 [-0.494, -0.322] [13]
**Non-Psychiatric Controls (N=1335) vs. Depression (N=179)**			
Hippocampus	0.074 [-0.027, 0.175]	3.51e-01	B: -0.144 [-0.225, -0.064] [14]
Amygdala	-0.096 [-0.197, 0.005]	2.28e-01	B: -0.060 [-0.132, 0.011] [14]
Lateral Ventricles	0.146 [0.045, 0.247]	6.73e-02	B: 0.056 [-0.017, 0.129] [14]
Posterior Cingulate	0.024 [-0.077, 0.124]	7.67e-01	R: -0.093 [-0.152, -0.034] L: -0.099 [-0.158, -0.040] [15]
Fusiform	-0.005 [-0.106, 0.096]	9.49e-01	R: -0.116 [-0.198, -0.033] L: -0.117 [-0.176, -0.058] [15]

Insula	-0.087 [-0.188, 0.013]	2.72e-01	R: -0.115 [-0.195, -0.035] L: -0.111 [-0.177, -0.045] [15]
Medial Orbitofrontal	-0.085 [-0.186, 0.016]	2.84e-01	R: -0.131 [-0.224, -0.039] L: -0.134 [-0.208, -0.059] [15]
Rostral Anterior Cingulate	-0.067 [-0.168, 0.034]	4.00e-01	R: -0.098 [-0.165, -0.031] L: -0.130 [-0.216, -0.044] [15]
**Non-Psychiatric Controls (N=1289) vs. Anxiety Disorder (N=155)**			
Putamen	-0.155 [-0.258, -0.051]	6.91e-02	R: -0.158 [-0.235, -0.080] L: -0.141 [-0.219, -0.062] [16]
Pallidum	-0.195 [-0.298, -0.091]	2.20e-02	R: 0.099 [0.021, 0.176] L: 0.129 [0.050, 0.208] [16]
Hippocampus	-0.024 [-0.127, 0.079]	7.78e-01	R: -0.060 [-0.137, 0.016] L: -0.084 [-0.161, -0.007] [16]
Amygdala	-0.187 [-0.291, -0.084]	2.79e-02	R: -0.040 [-0.118, 0.037] L: -0.076 [-0.153, -0.001] [16]

References:

1. Chen, T., & Guestrin, C. (2016). Xgboost: A scalable tree boosting system. In Proceedings of the 22nd acm sigkdd international conference on knowledge discovery and data mining (pp. 785-794).

2. Li, X., Morgan, P. S., Ashburner, J., Smith, J., and Rorden, C. (2016). The first step for neuroimaging data analysis: DICOM to NIfTI conversion. Journal of neuroscience methods, 264, 47-56.

3. Gopinath, K., Greve, D. N., Das, S., Arnold, S., Magdamo, C., and Iglesias, J. E. (2023). Cortical analysis of heterogeneous clinical brain MRI scans for large-scale neuroimaging studies. In International Conference on Medical Image Computing and Computer-Assisted Intervention, pages 35–45. Springer.

4. Billot, B., Greve, D. N., Puonti, O., Thielscher, A., Van Leemput, K., Fischl, et al. (2023). SynthSeg: Segmentation of brain MRI scans of any contrast and resolution without retraining. Medical image analysis, 86:102789.

5. Billot, B., Magdamo, C., Cheng, Y., Arnold, S. E., Das, S., and Iglesias, J. E. (2023). Robust machine learning segmentation for large-scale analysis of heterogeneous clinical brain MRI datasets. Proceedings of the National Academy of Sciences, 120(9):e2216399120.

6. Iglesias, J. E., Billot, B., Balbastre, Y., Magdamo, C., Arnold, S. E., Das, S., et al. (2023). SynthSR: A public AI tool to turn heterogeneous clinical brain scans into high-resolution T1-weighted images for 3D morphometry. Science advances, 9(5):eadd3607.

7. Dice, L. R. (1945). Measures of the amount of ecologic association between species. Ecology, 26(3), 297-302.

8. https://icd.who.int/browse10/2019/en

9. Desikan, R. S., Segonne, F., Fischl, B., Quinn, B. T., Dickerson, B. C., Blacker, D., et al. (2006). An automated labeling system for subdividing the human cerebral cortex on MRI scans into gyral based regions of interest. NeuroImage, 31(3):968–980.

10. Fischl, B. (2012). FreeSurfer. NeuroImage, 62(2):774–781.

11. Mackey, S., Allgaier, N., Chaarani, B., Spechler, P., Orr, C., Bunn, J., et al. (2019). Mega-analysis of gray matter volume in substance dependence: general and substance-specific regional effects. American Journal of Psychiatry, 176(2):119–128.

12. Van Erp, T. G., Hibar, D. P., Rasmussen, J. M., Glahn, D. C., Pearlson, G. D., Andreassen, O. A., et al. (2016). Subcortical brain volume abnormalities in 2028 individuals with schizophrenia and 2540 healthy controls via the ENIGMA consortium. Molecular psychiatry, 21(4):547–553.

13. Van Erp, T. G., Walton, E., Hibar, D. P., Schmaal, L., Jiang, W., Glahn, D. C., et al. (2018). Cortical brain abnormalities in 4474 individuals with schizophrenia and 5098 control subjects via the enhancing neuro imaging genetics through meta analysis (ENIGMA) consortium. Biological psychiatry, 84(9):644–654.

14. Schmaal, L., Veltman, D. J., van Erp, T. G., Sämann, P., Frodl, T., Jahanshad, N., et al. (2016). Subcortical brain alterations in major depressive disorder: findings from the ENIGMA Major Depressive Disorder working group. Molecular psychiatry, 21(6):806–812.

15. Schmaal, L., Hibar, D., Sämann, P. G., Hall, G., Baune, B., Jahanshad, N., et al. (2017). Cortical abnormalities in adults and adolescents with major depression based on brain scans from 20 cohorts worldwide in the ENIGMA Major Depressive Disorder Working Group. Molecular psychiatry, 22(6):900–909.

16. Groenewold, N. A., Bas-Hoogendam, J. M., Amod, A. R., Laansma, M. A., Van Velzen, L. S., Aghajani, et al. (2023). Volume of subcortical brain regions in social anxiety disorder: mega-analytic results from 37 samples in the ENIGMA-Anxiety Working Group. Molecular psychiatry, 28(3):1079–1089.
